# ROCKETS – a novel one-for-all toolbox for light sheet microscopy in drug discovery

**DOI:** 10.3389/fimmu.2023.1034032

**Published:** 2023-02-07

**Authors:** Joerg P. J. Mueller, Michael Dobosz, Nils O’Brien, Nassri Abdoush, Anna Maria Giusti, Martin Lechmann, Franz Osl, Ann-Katrin Wolf, Estibaliz Arellano-Viera, Haroon Shaikh, Markus Sauer, Andreas Rosenwald, Frank Herting, Pablo Umaña, Sara Colombetti, Thomas Pöschinger, Andreas Beilhack

**Affiliations:** ^1^ Interdisciplinary Center for Clinical Research Laboratory (IZKF) Würzburg, Department of Internal Medicine II, Center for Experimental Molecular Medicine, Würzburg University Hospital, Würzburg, Germany; ^2^ Pharmaceutical Research and Early Development, Roche Diagnostics GmbH, Penzberg, Germany; ^3^ Roche Pharmaceutical Research and Early Development, Roche Glycart AG, Schlieren, Switzerland; ^4^ Department of Biotechnology and Biophysics, Biocenter, University of Würzburg, Würzburg, Germany; ^5^ Institute of Pathology, University of Würzburg, Würzburg, Germany

**Keywords:** imaging, immunotherapy, preclinical drug development, biodistribution, cancer, light sheet fluorescence microscopy, EpCAM (CD326)

## Abstract

Advancing novel immunotherapy strategies requires refined tools in preclinical research to thoroughly assess drug targets, biodistribution, safety, and efficacy. Light sheet fluorescence microscopy (LSFM) offers unprecedented fast volumetric *ex vivo* imaging of large tissue samples in high resolution. Yet, to date laborious and unstandardized tissue processing procedures have limited throughput and broader applications in immunological research. Therefore, we developed a simple and harmonized protocol for processing, clearing and imaging of all mouse organs and even entire mouse bodies. Applying this Rapid Optical Clearing Kit for Enhanced Tissue Scanning (ROCKETS) in combination with LSFM allowed us to comprehensively study the *in vivo* biodistribution of an antibody targeting Epithelial Cell Adhesion Molecule (EpCAM) in 3D. Quantitative high-resolution scans of whole organs did not only reveal known EpCAM expression patterns but, importantly, uncovered several new EpCAM-binding sites. We identified gustatory papillae of the tongue, choroid plexi in the brain and duodenal papillae as previously unanticipated locations of high EpCAM expression. Subsequently, we confirmed high EpCAM expression also in human tongue and duodenal specimens. Choroid plexi and duodenal papillae may be considered as particularly sensitive sites due to their importance for liquor production or as critical junctions draining bile and digestive pancreatic enzymes into the small bowel, respectively. These newly gained insights appear highly relevant for clinical translation of EpCAM-addressing immunotherapies. Thus, ROCKETS in combination with LSFM may help to set new standards for preclinical evaluation of immunotherapeutic strategies. In conclusion, we propose ROCKETS as an ideal platform for a broader application of LSFM in immunological research optimally suited for quantitative co-localization studies of immunotherapeutic drugs and defined cell populations in the microanatomical context of organs or even whole mice.

## Introduction

Paradigm shifting mechanistic insights, conceptual advances, and compelling clinical outcomes have placed immunotherapy at center stage in the treatment of cancer patients. Direct targeting of cancer cells with therapeutic monoclonal antibodies (1–3), T cell-engaging antibody formats (4), antibody-drug conjugates (5, 6) and radioimmunotherapy (7), genetically modified chimeric antigen receptor (CAR) (8, 9) or T-cell receptor (TCR) T cells (10–12), and vaccination strategies (13–15) build an increasing armamentarium to treat cancer patients. Therapeutic approaches to indirectly boost the body’s natural defense against cancer have successfully improved clinical care by either targeting the cancer cells directly or the tumor microenvironment (3, 16) through blocking immune checkpoints (12, 17, 18) and activating preexisting endogenous immune effector mechanisms (19–21). To date, clinical success has cemented immunotherapy as a powerful pillar of modern cancer therapy. Yet, directing and taming the powers of an effective immune response against cancer cells remains challenging (22–25). Clearly, insights in the spatial organization of cancer, stroma and immune cell topography, distribution and the molecular regulation of potential therapeutic target antigens and the local and systemic regulation of immune effector mechanisms are key aspects determining success or failure of novel therapeutic strategies. Preclinical development requires careful consideration of tumor and tissue antigens as well as complex heterogeneous tumor microenvironments. Identifying promising targets implies to subsequently outweighing therapeutic benefits with potential toxicities, which remains a major challenge during preclinical and clinical development.

EpCAM (CD326) was one of the first human tumor-associated antigens (TAA) discovered with monoclonal antibodies more than forty years ago in patients with colorectal carcinomas (26). Since then it has become clear that many solid cancers of epithelial origin, such as colon, breast, pancreas and prostate carcinomas, aberrantly overexpress EpCAM. EpCAM fulfills many functions in the regulation of cell adhesion, proliferation, migration, stemness, and epithelial-to-mesenchymal transition (EMT) of carcinoma cells (reviewed in (27)). Notably, many healthy epithelial tissues also express EpCAM. However, healthy simple and pseudostratified epithelia in humans express EpCAM in basolateral membranes with the exception of hepatocytes and keratinocytes in contrast to the ubiquitous non-polarized overexpression profile in epithelial cancer cells (28, 29). These differential expression patterns have positioned EpCAM as an interesting antigen for targeted cancer therapy (27, 30) although EpCAM-targeted therapies must be closely assessed for on-target/off-tumor binding potentially resulting in adverse effects.

Therefore, advancing immunotherapies requires to further develop suitable tools and technologies to accelerate robust preclinical evaluation into successful clinical development.

Before entering clinical trials, any drug candidate must undergo extensive preclinical testing with the aim of predicting pharmacological properties and toxicological effects in humans (31). Herein, the *ex vivo* analysis of tissue specimens is often carried out based on histology. Modern histopathological analyses can rely on robust and highly standardized sample preparation techniques that have evolved over decades. However, it has been demonstrated that thin sections of embedded tissues are not always representative for the entire specimen (32, 33). Furthermore, creating hundreds of physical sections is extremely time consuming, laborious and uses up the specimen for further analysis, especially when rare events need to be detected within large tissue specimens (34–36).

Over the last two decades, light sheet fluorescence microscopy (LSFM) has emerged as a non-destructive technology offering rapid high-resolution imaging by creating optical sections of large intact tissue specimens (37). Consequently, LSFM has been applied across many fields of research (38) like developmental biology (39–43), neurobiology (44–47), cancer research (48–51) and immunology (52–56). High acquisition rates and recent progress towards batch-wise imaging of multiple specimens render LSFM principally suitable for large-scale preclinical studies with dozens or even hundreds of samples. However, as a prerequisite for mesoscopic LSFM imaging, specimens must be rendered optically transparent (clearing) (57–59). Clearing is generally achieved by removing light-absorbing components from the tissue and reducing scattering through the homogenization of different refractive indices (RI) (58). Many protocols have emerged for the clearing of murine and human tissues, but most published procedures are limited to processing few specimens at a time and are often tailored for a specific tissue of interest (60, 61). For some tissues like the small and large intestine, no procedures exist that enable clearing of entire organs, rather than small segments (52, 62–65). Additionally, almost all protocols for murine tissues require animals to be perfused, a laborious and time-consuming procedure to flush out the blood from animals (66). Due to these limitations experimenters face great complexity if they want to clear more than one type of tissue or many specimens in parallel. Therefore, sample preparation still obstructs LSFM-based studies in preclinical drug development.

To this end we report three advances to overcome current challenges to routinely apply LSFM for advancing novel immunotherapy strategies. First, we combined and harmonized a clearing procedure of murine specimens optimally suited for standardized and high-throughput LSFM. Our *Rapid Optical Clearing Kit for Enhanced Tissue Scanning* (ROCKETS) approach, which does not require transcardial perfusion, combines in the first step hydrophilic expansion (hyperhydration), delipidation and decolorization and in the second step dehydration and organic solvent-based RI-matching. Second, we developed a technique for LSFM analysis of the entire gastrointestinal tract (GIT), which we termed 3D-Swiss Rolls technique. Third, we demonstrate that ROCKETS is also suited for LSFM of whole mouse bodies.

Finally, we investigated the biodistribution of an EpCAM-specific antibody employing ROCKETS and semiquantitative LSFM imaging, which resulted in unanticipated outcomes.

On top of confirming well-recognized sites of EpCAM expression we report, to our knowledge for the first time, accentuated EpCAM expression at all types of gustatory papillae of the tongue and especially in the choroid plexi in brain ventricles as well as the duodenal papillae. Postmortem stainings on paraffin embedded tissue samples could independently confirm these findings, even on human tongue and intestinal specimens. We deem our observations as highly relevant to be considered for cancer immunotherapeutic approaches. In summary we propose ROCKETS combined with LSFM to complement current immunohistochemical analyses for large-scale assessment of *in vivo* drug development to advance immunotherapy.

## Methods

### Animal models, handling and care

Female ten-week-old C57BL/6 inbred mice were obtained from Charles River Laboratories Germany GmbH, Sulzfeld, Germany. Studies were approved by the Government of Upper Bavaria (Regierung von Oberbayern, Munich, Germany; ROB-55.2-2532.Vet_02-19-5) and in accordance with the European directive 2010/63/EU for animal research. For subcutaneous tumors, 3*10^5^ murine pancreatic ductal adenocarcinoma cells KPC-4662wt were applied as suspension in 100 µl matrigel (50% [v/v]), FisherScientific, Corning™ 354234) into the right flank of the animals. Animals were euthanized by cervical dislocation for whole-organ analyses.

### Administration of conjugated antibodies

20 µg of anti-mouse EpCAM (CD326) antibody, conjugated with AlexaFluor750 (R&D Systems, FAB8998S, clone G8.8R), was administered to mice intravenously (i.v.) into the tail vein 24 hours (h) before euthanasia.

### Fixation of organs

Tissues of interest were excised immediately after euthanasia and rinsed briefly with deionized water (dH20) to remove hair or body fluids. Specimens were transferred to histological cassettes (Simport, Macrosette M512) for fixation. Tissues of the small and large intestines were processed according to the 3D-Swiss Roll procedure (below). All tissues were fixed using neutral buffered formaldehyde solution (NBF, 4% formaldehyde, VWR Chemicals 9713.9025) of at least ten times the volume of the dissected specimens for 14 to 18 h at 4°C with gentle agitation in the dark.

### Preclearing and *ex vivo* immunofluorescence stainings

For clearing of blood-rich and large mouse organs without transcardial perfusion, light-absorbing and -scattering tissue components were chemically removed by immersion in a preclearing reagent before dehydration and organic solvent-based RI matching:

Fixed tissues were incubated at 30°C in a minimum of 15 ml per whole organ with gentle agitation in the dark for 2 to 4 days (d), with one exchange after 2 d. The preclearing reagent comprised 20% (v/v) Quadrol^®^, (N,N,N′,N′-Tetrakis(2-Hydroxypropyl)ethylenediamine, CAS102-60-3, Sigma Aldrich 122262-1L), 10% (v/v) TWEEN-80^®^ (Polyethylene glycol sorbitan monooleate, CAS 9005-65-6, Sigma Aldrich P1754-500ML), 10% (v/v) TEA (2,2′,2′′-Nitrilotriethanol, CAS 102-71-6, Sigma Aldrich 90279), 10% (v/v) DMSO (Dimethyl Sulfoxide, CAS 67-68-5, Sigma Aldrich D5879-500ML), 10% (w/v) urea (CAS 57-13-6, Sigma Aldrich U5378-1KG) dissolved in dH_2_O. For mixing ~100 ml/l of dH_2_O was applied before adding other components. Quadrol^®^ was heated up to ~40°C to reduce viscosity and enable pouring.

After preclearing of organs, the preclearing reagent was removed, specimens were rinsed briefly with dH_2_O and then washed with PBS_PC_ (phosphate-buffered saline with added biocide ProClin300, Sigma-Aldrich 48912-U, at 0.05% v/v) four times for 1.5 h, once overnight and again twice for 1.5 h at room temperature (RT) before proceeding to dehydration. At this step, samples could be stored in PBS_PC_ at 4°C in the dark for up to four weeks without significant loss of fluorescence signals. Preclearing of non-perfused organs improved imaging for all tissues and was indispensable for the following organs (incubation time/notes): spleen (4 d), kidneys (4 d), liver (4 d), heart (4 d, coronal section exposing all four chambers of the heart using a scalpel), large/blood-rich tumors (4 d, larger than 500 µm in diameter), tongue (4 d), lungs (2 d), thymus (2 d).

For *ex vivo* immunofluorescence stainings of murine lymph nodes (LN), we incubated tissue specimens overnight in 1.4 ml blocking solution at 30°C to prevent nonspecific binding. The blocking cocktail comprised 0.3% Triton X-100, 10% DMSO, 2% normal mouse serum, 2% normal rat serum, all (v/v%), and 2% BSA (bovine serum albumin, w/v), dissolved in PBS_PC_. For staining, 700 µl of the blocking buffer was removed and replaced with Ventana Antibody Diluent (Roche Diagnostics AG, Art.05261899001) containing 0.3% Triton X-100. To this solution, antibodies were added at 10 µg/ml: anti-CD3, conjugated with AlexaFluor594 (clone 17A2, Biolegend, Cat. 100240) and anti-CD19, conjugated with AlexaFluor647 (clone 6D5, Biolegend, Cat. 115522). Lymph nodes were then incubated at 30°C for 3 d with gentle agitation in the dark before being washed multiple times for 1 h and again overnight in PBS_PC_ before being processed for clearing and LSFM imaging.

For staining of cell nuclei within mouse tissues, excised tumor specimens were incubated with 5 μg/ml propidium iodide (PI), dissolved in the preclearing reagent, for 4 d at 30°C and gentle agitation. The preclearing reagent was removed and rinsed off using PBS_PC_ and samples were washed twice in PBST_PC_ (PBS + 0.1% ml Tween-20+ProClin300) and again overnight. On the next day, samples were washed again in PBS_PC_ for 2 h before being processed for clearing and LSFM imaging.

For immunofluorescence stainings of mouse organs, tissues were fixed as described above and dehydrated using EtOH (Carl Roth, Cat. 0911) in an automated tissue processor (Tissue-Tek VIP^®^ 6 AI Vacuum Infiltration Processor, Sakura) inside histology cassettes, washed once with xylene (Roth, Cat. 9713.3) for 20 minutes and paraffinized using the automated tissue processor. Paraffinized specimens were embedded in blocks, cooled overnight, and cut into 2.5-5 µm thick slices using a rotary microtome HM355S heavy duty with section transfer system and Cool-Cut module (Thermo Scientific). Floating tissue slices were mounted on glass slides and dried at 30°C overnight. Sections were then deparaffinized and rehydrated automatically using Gemini AS Automatic Stainer programmed to apply: 3x xylene (3 min), 2 x 100% ethanol (2 min), 95% ethanol (1 min), 70% ethanol and dH_2_O (1 min). Antigen retrieval was performed by boiling sections in a steamer in Discovery CC1 solution (Ventana, 950-500) for 30 min. To prevent unspecific binding, tissue sections were blocked using protein block solution (Dako, X0909) for 10 min at RT. All tissues except brains were directly incubated with 10 µg anti-mouse EpCAM antibody conjugated with Alexa Fluor 647 (Biolegend, Cat. 118211, clone G8.8) diluted in antibody diluent (Ventana, 251-018) for 1 h at RT.

For murine brain sections, antibody-blocking was performed as reported by Rogers et al., 2006 (67), by coincubating 25 µg of the antibody with 30 mM of L-reduced glutathione (Sigma Aldrich, 70-18-8) diluted in 1X TBST (G-biosciences, R042) on ice for 1 h before applying to sections for 1 h at RT as well. After washing, nuclei were counterstained and mounted with Fluoro-Gel II mounting medium with DAPI (Electron Microscopy Sciences, 17985-51), and sections were left to dry until the following day. The slides were imaged using slide scanner AxioScan^®^ 7 (Carl Zeiss, Jena, Germany), and files were exported and visualized using Imaris Software, version 9.9.1.

Human FFPE tissues were stained using fully automated tissue stainer Leica Bond-III (Biosystems Switzerland), pretreated with citrate buffer at pH 6, and anti-EpCAM antibody (clone MOC31, Cell Marque, 1:200).

### 3D-Swiss Rolls for specimens of the GI tract

For specimens of the small and large intestine, the GIT was removed as a whole from the abdominal cavity by cutting the distal esophagus (approx. 3-5 mm from the stomach) and the rectum. Attached mesentery was removed by careful pulling with forceps or cutting. If the pancreas was to be analyzed as a whole, the GIT was removed together with the entire pancreas and the spleen and then separated ex situ as a whole. Subsequent intestinal incisions were made at the pyloric sphincter, the ileocecal valve and distal of the cecocolic orifice, thereby separating stomach, small intestine, cecum and colon. Stomach and cecum were transferred to ice-cold PBS_PC_ to slow down autolytic processes while the colon and small intestine were processed.

The distal end of the small intestine was gently pulled onto a rodent oral feeding gavage with ball-tip, attached to a 50 ml syringe containing ice-cold PBS_PC_. Holding the sample firmly on the gavage with fingers, chyme and feces were flushed out with ice-cold PBS_PC_. While flushing, the specimen was gradually and gently pulled onto the gavage to allow for thorough removal of feces also from the proximal end. After rinsing, small intestines were immediately flushed again and filled with NBF using a separate syringe with a feeding gavage. The small intestines were then laid out flat, forming an “N”, and cut into three equally long sections (SI 1-3). The created segments were transferred to a beaker containing NBF, noting the correct order of segments as well as the proximal and distal ends. The colon was processed as one single specimen.

3D-Swiss Rolls were formed as quickly as possible to prevent specimens from becoming too rigid for proper rolling. Rolling of the colon was conducted first, as it became too rigid for rolling shortly after immersion in NBF. To create 3D-Swiss Rolls, the specimens were cut open longitudinally along the mesenteric line and then transferred back into a flat dish containing NBF. Using forceps, the specimens were gently pulled over a wooden tampone swab (LP Italiana, 112298, cotton ends removed) with the luminal side facing outward and starting with the proximal end of the colon. Once rolled, the end of the swab was placed into the lower corner of a large sample-processing cassette (Macrosette M512, Simport) and the protruding end cut off at the opposite corner of the cassette. This way, specimens could be placed diagonal in the cassette without touching the surface of the cassette (thus avoiding imprints in the tissue after fixation). The cassette was immediately transferred to NBF for fixation. Each section of the SI was processed as described for the colon, except rolling was started at the distal end of each segment to avoid excessive squeezing of the longer proximal villi. Stomach and cecum were then cleaned by inserting a gavage needle attached to a syringe containing ice-cold PBS_PC_ into the stomach through the pylorus or cecum through the ileocecal valve, respectively. Contents were flushed out until organs were empty and rinsing buffer was clear. The specimens were filled with NBF and transferred into a flat histological cassette with a paper inlay for fixation in their physiological shape. In general, complete removal of chyme or feces from all the specimens is important because the plant-based nutrition of mice shows very high autofluorescence in LSFM imaging. At the same time, quick processing is even more essential during dissection to prevent autolytic damage of the tissues. Therefore, if not all residues of chyme or feces could be removed during dissection, further cleaning could be conducted after fixation.

After fixation, 3D-Swiss Roll samples were unraveled from wooden holding sticks in a large bowl containing ice-cold PBS_PC_ and remaining chyme and feces were carefully removed. Specimens were then rolled up again in the same orientation, now on plastic stirring spatulas (Brand, VWR 441-0217). This was necessary because the wooden cotton swabs used during dissection left dark marks on the tissues upon dehydration. Rolled specimens were transferred back to cassettes for dehydration and clearing. Specimens were then dehydrated following the automated procedure described below. After dehydration, plastic stirring rods were removed before immersion in BABB because polystyrene does not withstand organic solvents.

### Processing of whole mice

For clearing of entire mouse bodies, animals were euthanized by CO_2_ inhalation and immediately perfused transcardially with 40 ml PBS with 10 IU Heparin (B. Braun, 25.000 IE/5 ml Heparin sodium, Melsungen, Germany) at 2 ml/min, immediately followed by perfusion with 60 ml NBF with 10 IU Heparin at 1.5 ml/min and again 20 ml PBS with 10 IU Heparin sodium at 2 ml/min to remove NBF. Mice were then decalcified by constant perfusion and immersion in 20% EDTA solution (Entkalker Soft, Carl Roth 6484.2) for six days at 2 ml/min in the dark. EDTA was removed by rinsing and perfusion with dH_2_O for 30 min. The skin was removed and the GIT cleaned by incising at multiple locations and rinsing out contents with dH_2_O using a syringe with an oral feeding gavage. Mice were then immersed in the preclearing reagent at 30°C for 14 d with gentle agitation and exchanges of the reagent after three, six and nine days. To control evaporation, incubation was carried out in a container with airtight lid. After the last step, the preclearing reagent was discarded and animals briefly washed in PBS_PC_ to remove bulk residues of the reagent. Mice were then washed in PBS_PC_ for a total of 24 h: 3x 3 h, overnight and again 2 h before proceeding to methanol- (MeOH-)based dehydration, delipidation and RI matching.

### Dehydration and refractive index matching (clearing)

Specimens were dehydrated using two different procedures, depending on the tissue. All individual organs except the brain were dehydrated using EtOH (Carl Roth, Cat. 0911), an automated tissue processor (Tissue-Tek VIP^®®^ 6 AI Vacuum Infiltration Processor, Sakura) inside histology cassettes. The custom protocol comprised of eight steps of 30 min each in a low-pressure environment to enhance diffusion of an increasing concentration series of EtOH: 70%, 70%, 80%, 80%, 90%, 90%, 100%, 100% (v/v). After dehydration, cassettes were dried using a paper cloth before RI matching by immersing specimens in BABB (one part benzyl alcohol [BA, Sigma Aldrich 305197-2L] and two parts benzyl benzoate [BB, Merck Millipore 8187011000]). Specimens were incubated in BABB in the dark for 24 h until fully transparent (less for very small or permeable tissues, two days for whole mice). Once cleared, specimens could be stored light-protected for at least three months at 4°C without loss of fluorescence signals.

Whole brains and whole mice were dehydrated manually at room temperature (RT) using methanol (MeOH, Merck Millipore 1060092511) and additionally delipidated with dichloremethane (DCM, Merck Millipore 1006681000). Brains were dehydrated at RT with gentle agitation in the dark at 20%, 40%, 60%, 80% and 100% methanol (v/v, diluted with dH_2_O, 1.5 h each) and again in fresh 100% methanol overnight at 4°C. Delipidation was carried out in 66% (v/v) DCM and 33% MeOH for 5 h at RT with gentle agitation and specimens briefly washed in 100% DCM for 15 minutes before immersion in BABB until fully cleared.

Whole mice were processed according to the same protocol but with longer incubation times of 4 h for the first two incubations (20%, 40% methanol), o/n (60% methanol), 8 h (80%), o/n (2 x 100% methanol and DCM/methanol) and 30 minutes (100% DCM). As higher concentrations of methanol evaporated more quickly, specimens were incubated in airtight glass containers.

### Light sheet fluorescence microscopy, data conversion and visualization, scoring

Imaging was conducted using either a light sheet fluorescence microscope (LSFM) Ultramicroscope II^®^ (UM2, LaVision Biotec, Bielefeld, Germany; now part of Miltenyi Biotec, Bergisch Gladbach, Germany) or LSFM Ultramicroscope Blaze^®^ (UM Blaze, Miltenyi Biotec, Bergisch Gladbach, Germany). The UM Blaze was equipped with an edge 4.2 sCMOS (2048 x 2048 active pixels, pixel size 6.5 µm) camera (PCO Instruments). The UM2 instrument was modified compared to the original model at the excitation light path: Instead of six light sheets for excitation of fluorescence, the illumination light was channeled in only two light sheets that were oriented opposite towards each other. The UM2 was equipped with an Andor Neo 5.5 sCMOS (2560 x 2160 active pixels, pixel size 6.5 µm) camera (Oxford Instruments). The thickness of the generated light sheet is estimated at 6 µm at its thinnest point, based on the chosen numerical aperture setting of 0.02 of the sheet optics for both instruments. As light source, a supercontinuum white light laser SUPERK extreme EXR-20 (NKT Photonics) with a maximal power output of 2 W was applied combined with optical bandpass filters for fluorescence excitation and emission detection. For acquisition of anatomy using the UM2, tissues were imaged by excitation at 545 nm with a filter bandwidth of 20 nm and emission was detected at 595 nm with a filter bandwidth of 40 nm (545 (20)nm → 595(40)nm), the anti-EpCAM IgG2a conjugated with AlexaFluor^®^750 was detected at 747(33)nm → 786(22)nm. Using the UM Blaze, anatomy was acquired at 520(40)nm → 572(23)nm and the antibody was detected at 740(40)nm → 824(55)nm. For imaging of entire mice, mosaic scans (comprising eight individual z-stacks per channel) were conducted using the built-in feature of the UM Blaze. Image acquisition for an entire mouse body took 4-5 h. Subsequent image stitching was conducted using the Imaris Stitcher version 9.3.1 (Oxford Instruments, United Kingdom). Raw image data in the.tiff file format was converted to the native Imaris file format using the Imaris file converter version 9.3 or higher and visualizations were created using Imaris version 9.5 or higher (Oxford Instruments, United Kingdom). Scoring of binding was conducted semi-quantitively by comparing maximum fluorescence signal intensities of each tissue in whole mouse scans of three mice.

### Processing of cleared tissue specimens for conventional 2D histology and slide scanning

Histological assessment after 3D-LSFM imaging was conducted by removal of BABB and washing with xylene (Roth, Cat. 9713.3) for 10 min before paraffinization using a Tissue-Tek VIP 6 Vacuum Infiltration Processor (Sakura). Paraffinized specimens were cut to 2.5 µm thick slices using a rotary microtome HM355S heavy duty with section transfer system and Cool-Cut module (Thermo Scientific). Floating tissue slices were picked up on glass slides and dried at 30°C o/n. Tissue sections were then deparaffinized and rehydrated automatically using a BenchMark ULTRA autostainer (Roche Ventana) programmed to apply: 3x xylene (3 min.), 2x 100% ethanol (2 min), 95% ethanol (1 min.), 70% ethanol and dH2O (1 min.). Hematoxylin and Eosin (H&E) stainings were performed using a VENTANA HE 600^®^ system (Roche). The slides were imaged using slide scanner AxioScan^®^ 7 (Carl Zeiss, Jena, Germany) and files were exported and visualized using ZEN Blue Edition Software, version 2.3 (Zeiss).

## Results

### ROCKETS toolbox for passive clearing of mouse tissues

**Figure 1:**
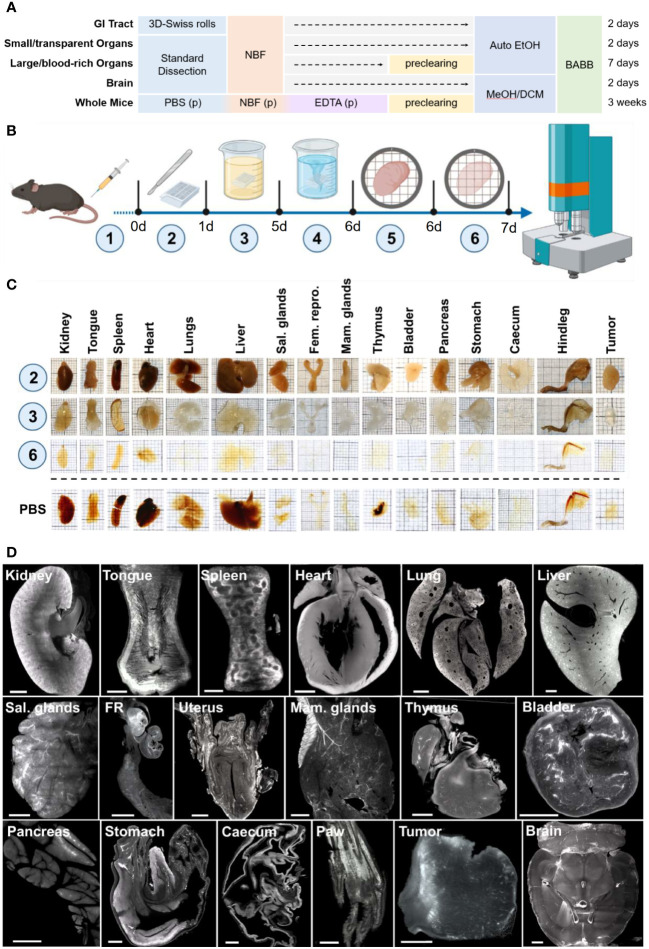
Modular clearing approach of the ROCKETS processing toolbox allows for simplified sample preparation for LSFM imaging. **(A)** Overview of presented procedures for processing and simplified clearing of mouse tissues or whole mouse bodies. GITs are processed using the 3D-Swiss Rolls procedure prior to fixation to enable holistic imaging. Other internal organs and tissues can be processed according to size and blood content. Non-perfused large and blood-rich tissues are precleared using the developed preclearing reagent before dehydration. Smaller tissues with less blood content do not require preclearing. All tissues except for the brain and whole mice are dehydrated with ethanol using an automated vacuum tissue processor. Due to its high lipid content, the brain is dehydrated in methanol and additionally delipidated using dichloromethane (MeOH/DCM). Only whole mice require perfusion to ensure timely fixation and decalcification of bones before the preclearing step. All specimens are cleared (RI matching) and imaged in BABB. Indicated times are total processing times from the day of dissection to cleared specimens. (p) = perfusion. **(B)** Workflow of passive preclearing of non-perfused murine tissues. Fluorescence-labeled molecules are applied *in vivo* (1) prior to euthanasia, tissue dissection and fixation overnight (2). Fixed specimens are incubated in the ROCKETS preclearing reagent (3) and washed with PBS_PC_ (4) before transfer to vacuum-enhanced dehydration (5) and RI matching with BABB (6). **(C)** Photographs of mouse tissues at indicated step of preclearing. Specimens are opaque and still contain blood pigments after fixation (2). After preclearing (3) samples are fully decolorized and swollen and become completely transparent after dehydration and RI matching (6). The bottom row shows tissues after dehydration and RI matching without preclearing (immersed in PBS). Particularly blood-rich organs are insufficiently cleared without perfusion or preclearing. Thick squares of the grid = 5 mm. **(D)** Maximum intensity projections (MIPs) of LSFM images (z = 50 µm) of the tissue’s autofluorescence (545 nm → 595 nm) at the widest diameter of precleared tissues. FR = Female reproductive organs (oviduct and ovary), Sal. glands = Salivary glands. All tissue areas could be imaged entirely without blurring. *brain was not precleared, but dehydrated and delipidated using MeOH and DCM. Scale bars = 1 mm.

To develop a simple and harmonized protocol for large-scale and high-throughput LSFM for immunological research and preclinical drug development of all mouse organs or whole bodies for LSFM imaging we integrated, adapted, and complemented existing procedures for various tissues. To this end we focused on passive clearing techniques, which are often categorized by hydrophilic and organic solvent-based approaches (66, 68–70) We chose to combine these concepts in a coherent two-step procedure, which we subsequently termed Rapid Optical Clearing Kit for Enhanced Tissue Scanning (ROCKETS). First, hydrophilic expansion (hyperhydration), delipidation and decolorization similar to the previously published CUBIC by Susaki et al. in 2014 (71) and, second, dehydration and organic solvent-based RI matching as described already in 1914 by Werner Spalteholz (72), which was first applied for modern LSFM of biological tissues by Hans-Ulrich Dodt et al. in 2007 (40) and later refined in the DISCO-family of clearing protocols, initiated by the work of Ali Ertürk et al. in 2012 (73–75). Subsequently, our developed ROCKETS toolbox allowed for choosing to process particular or all tissues of interest or even whole mice (**Figure 1A**), for which each critical step is outlined below.

### Fixation

Tissue fixation in general is an important factor in tissue processing that has rarely been considered for tissue clearing. After *in vivo* i.v. administration of fluorescently labeled antibodies and euthanizing mice we fixed tissues using neutral buffered formalin (NBF), which covalently cross-links proteins (76) to keep bound antibodies linked to their target. We observed that the duration and temperature of fixation in NBF had a significant impact on clearing performance and undesired autofluorescence. Over-fixation (>12 h at RT or >24 h at 4°C) led to insufficient clearing, particularly of large and blood-rich tissues as well increased background fluorescence. Fixation <8 h at RT or <12 h at 4°C for large organs resulted in tissue damage during subsequent processing steps and lower specific fluorescence signal intensities in affected tissue regions. Thus, whole organs were fixed in NBF overnight at 4°C for 14-18 h immediately after dissection.

### Passive preclearing and immunofluorescence and nuclear stainings

The goal of any tissue clearing protocol is to maximize transparency through reducing light absorbance and scattering (58, 69). The major source of absorbance in most biological tissues is hemoglobin, the pigment of red blood cells (66). Therefore, to flush blood from the vessels most clearing protocols start with transcardial perfusion of mice, a laborious and messy procedure (77).

To enable passive clearing and omit perfusion, we developed the concept of a hydrophilic preclearing step prior to dehydration and organic solvent-based RI matching (**Figures 1B, C**). We reasoned that the original CUBIC cocktail as published by Susaki and colleagues in 2014 (71) and in particular the amino alcoholic component Quadrol^®^ (*N,N,N′,N′-Tetrakis(2-Hydroxypropyl)ethylenediamine*) should be principally suitable to omit perfusion. The decolorizing ability of Quadrol^®^ is based on releasing the light-absorbing prosthetic heme from erythrocytes (69). We found that the decolorizing effect for fixed whole liver lobes treated with various dilutions of Quadrol^®^ generally increased with increasing concentrations, peaking at approximately 20% (v/v, in dH_2_O) above which we observed no further improvement in effect nor time. Starting from the decolorizing reagent we rationally added further components to the mixture to reduce scattering and increase permeability. The cause for light scattering in biological tissues are inhomogeneous refractive indices, particularly between aqueous compartments, proteins, lipids and fatty acids (58). To elute different types of fats (delipidation) we added two surfactants, Tween-80^®^ (T-80) and triethanolamine (TEA) at 10% (v/v), thereby avoiding commonly used octylphenol ethoxylates like Triton™ X-100, which have been banned from using in the European Union by the European Chemicals Agency (ECHA) due to environmental toxicity. We further included urea as applied in the CUBIC reagent, which induces hyperhydration and corresponding swelling of the tissues, thereby increasing molecular flux and facilitating diffusion of all components through the tissues (69). As described previously for brain tissue (45) we also observed increased swelling of all organs with higher urea concentrations (not shown). At concentrations above 15% urea (w/v) we observed macroscopic deformations of large organs such as the liver (**Suppl. Figure S1**). These morphological changes were permanent and not reversed through dehydration (and resulting shrinkage) and clearing. Therefore, we added urea at 10% (w/v), which was sufficient to induce reversible swelling without affecting anatomy. Dissolving of all components in deionized water (dH_2_O) yielded a highly viscous solution. To reduce viscosity, we incubated specimens at 30°C and added 10% dimethylsulfoxide (DMSO), which is known to promote both hydrophilic and lipophilic permeation through tissues (78). The final cocktail, which we termed *preclearing reagent*, was a yellowish solution with water-like viscosity at 30°C.

Non-perfused mouse organs were incubated in 15 ml preclearing reagent per whole organ for two to four days at 30°C, depending on the organ. After treatment, all tissues except bone marrow appeared completely colorless, swollen and partially transparent (**Figure 1C**, step 3). We detected splenic melanosis in some specimens, presenting as dark spots at one end of the spleen, as is frequently observed in mice with dark coat color (79) and which could not be removed through preclearing. After washing, specimens appeared with a yellow-whitish non-transparent color and had re-gained their physiological size.

After preclearing, we dehydrated and cleared specimens using a 1:2 (v/v) mixture of benzyl alcohol and benzyl benzoate (BABB, see below). After RI matching, all organs were fully transparent when treated using the preclearing reagent (**Figure 1C**, step 6). We confirmed the preservation of the microanatomical integrity of all major organs after preclearing, dehydration and RI matching, with subsequent hematoxylin and eosin (H&E) stainings of sections of processed tissues (**Suppl. Figure S2**). In LSFM scans of the autofluorescence at 545 → 595 nm precleared tissues could be imaged at high resolution throughout their entire volume (**Figure 1D**) and showed overall higher fluorescence intensities compared to non-treated samples. At low wavelengths and in deep regions of certain tissues such as the liver images blurred and showed an inhomogeneous illumination in the center of the samples (**Suppl. Tab. S1**). However, at excitation wavelengths of 545 nm or higher, tissues could be imaged with high contrast and no blurring at full depth and with homogeneous illumination when preclearing was conducted before RI matching. As the only exception, the liver remained inhomogeneously illuminated even between 545 nm → 595 nm excitation as a result of light absorbance. However, optimal and homogenous illumination could be achieved in the liver at 680 nm and higher (**Suppl. Figure S3**). Without preclearing, particularly large organs still contained significant amounts of blood and appeared generally more opaque after dehydration and RI matching (**Figure 1C**, PBS). Without the preclearing steps, several organs (kidneys, tongue, spleen, heart, lungs, liver, thymus, hindleg) could not be imaged entirely, particularly at lower wavelengths due to light attenuation and blurring towards the center (not shown). However, smaller organs or tissues with lower vascularization such as cecum, stomach, female reproductive tract, bladder, and LN were sufficiently transparent without preclearing. Thus, we concluded that preclearing of these organs could be omitted if imaging at higher wavelengths is intended (**Suppl. Tab. S1**). Yet, importantly, preclearing also improved image quality and signal-to-noise ratio (SNR) for small organs.

To demonstrate the general compatibility of the clearing procedures with *ex vivo* immunofluorescence stainings we incubated a LN in a solution containing an AlexaFluor 594-labeled anti-CD3 antibody and an AlexaFluor647-labeld anti-CD19 antibody before conducting the ROCKETS clearing procedure. Clearly visible B cell follicles and T cell zones confirmed successful staining of the LN (**Suppl. Figures S4A, B**). Lastly, we tested whether nuclear stainings, as applied on a routine basis in histology, can be conducted also for LSFM. Therefore, we stained an explanted murine tumor sample by adding propidium iodide to the preclearing reagent and detected individual cell nuclei throughout the specimen (**Suppl. Figures S4C–E**).

### 3D-Swiss Rolls for holistic assessment of the gastrointestinal tract

**Figure 2:**
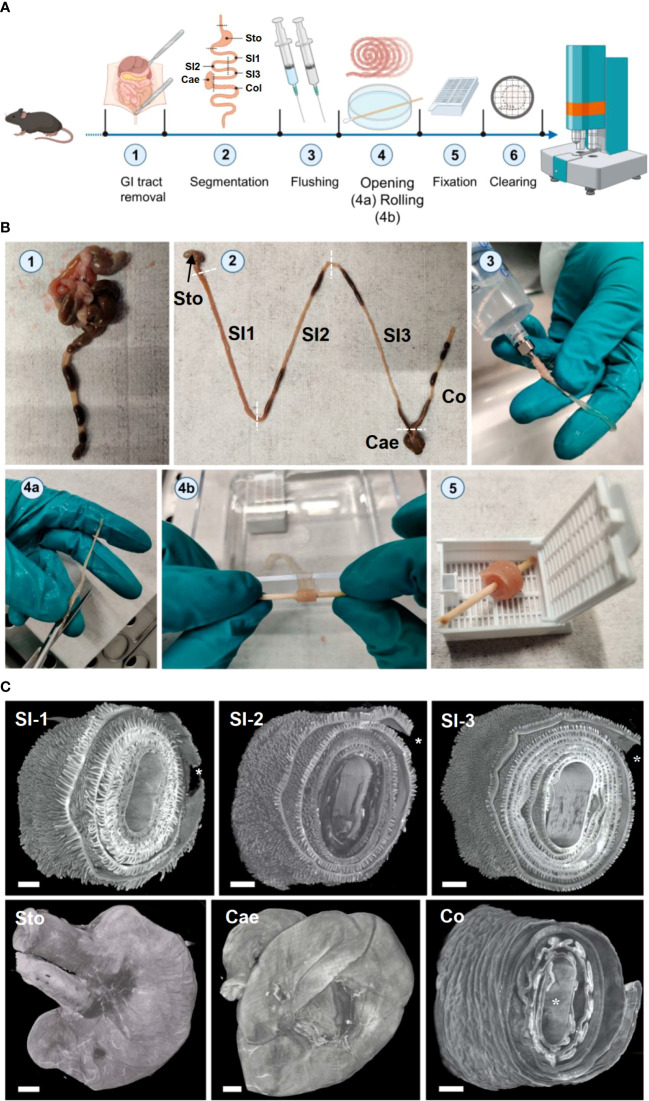
3D-Swiss Rolls sample preparation procedure for LSFM imaging enables holistic assessment of the entire GIT. **(A)** Schematic and **(B)** photographic representation of the 3D-Swiss Rolls workflow. (1) After euthanasia the lower GIT is disconnected from the body by incisions at the esophagus and rectum and removed entirely. (2) Six specimens are created by cutting as indicated by dashed lines: stomach (STO), three segments of the small intestine (SI 1-3), cecum (CAE) and colon (COL). (3) Each specimen is cleaned by flushing out chyme and feces with PBS_PC_ and then immediately filled with NBF for fixation. (4a) SI and COL segments are cut open along the mesenteric line and (4b) rolled up on wooden sticks to create 3D-Swiss Rolls. (5) The created 3D-Swiss Rolls are then fixed without touching the surfaces of the histology cassette for 14-18 h in NBF at 4°C. After fixation, 3D-Swiss Rolls are unwound and re-rolled on plastic stirring rods for dehydration and clearing (not shown). **(C)** Surface rendering of LSFM image stacks of the tissue autofluorescence (545 → 595 nm, grey). 3D-Swiss Roll segments of the small intestine (SI1-3) and colon (Col). Stomach (Sto) and cecum (Cae) retained their physiological form. *Proximal end of the organ in 3D-Swiss Rolls. Scale bars = 1 mm.

The sheer size and the convoluted tubular structure of the GIT, particularly the small and large intestine, makes it difficult to investigate microscopically. The GIT is neither structurally nor functionally a homogeneous tissue and it is therefore important to analyze it as a whole (80, 81). Therefore, we adapted the histological preparation technique of *Swiss rolling* (82–84)) for LSFM-based three-dimensional imaging of the GIT. In reference to this, we termed the samples created by our technique *3D-Swiss Rolls*. After euthanizing mice, we removed the lower GIT as a whole and separated it *ex situ* into six specimens (**Figure 2**, steps 1, 2): Stomach (STO), three equally long segments of the small intestine (SI 1-3), cecum (CAE) and colon (COL). Using an oral feeding gavage needle connected to a syringe, we flushed out chyme and feces and immediately filled the specimens with NBF (**Figure 2**, step 3) to accelerate fixation and prevent autolytic processes. We used NBF instead of acidic Bouin’s fixative (as applied in the original procedures) to avoid fluorescence quenching and to streamline the workflow with processing of other organs. Next, we cut open the small intestine and the colon longitudinally (**Figure 2**, step 4a) and rolled up the segments on wooden sticks with the luminal side facing outward and further fixed them in this position (**Figure 2**, steps 4b and 5). Quick processing turned out as essential during all steps of the procedure. If not processed quickly, particularly the stomach and the proximal third of the small intestine started to deteriorate within minutes due to autolytic processes from exposure to gastric acid, bile and digestive enzymes as observed previously (85). Otherwise, resulting damages to the tissues’ microanatomy due to slow tissue processing might be misinterpreted for toxicity-related effects of investigated drugs. Also, 3D-Swiss Rolls had to be placed carefully into histology cassettes without being pressed against the surface to avoid imprints on the specimens (**Suppl. Figure S5**). Once fixed, the rolls could be handled with less caution and retained their rolled form during washing, change of holding sticks and automated dehydration. After dehydration and RI matching, samples were stiff and could be easily mounted for LSFM imaging. We confirmed the anatomical integrity of the GIT specimens by LSFM imaging as well as slide-based histology with hematoxylin and eosin (H&E) staining (**Suppl. Figures S6A**, **S7**). High-resolution LSFM of 3D-Swiss Rolls allowed us to identify individual cells (enterocytes, goblet cells and Paneth cells) and single nuclei in the entire GIT without additional counterstaining (**Suppl. Figures S6A, C**).

### Clearing of whole mouse bodies

**Figure 3:**
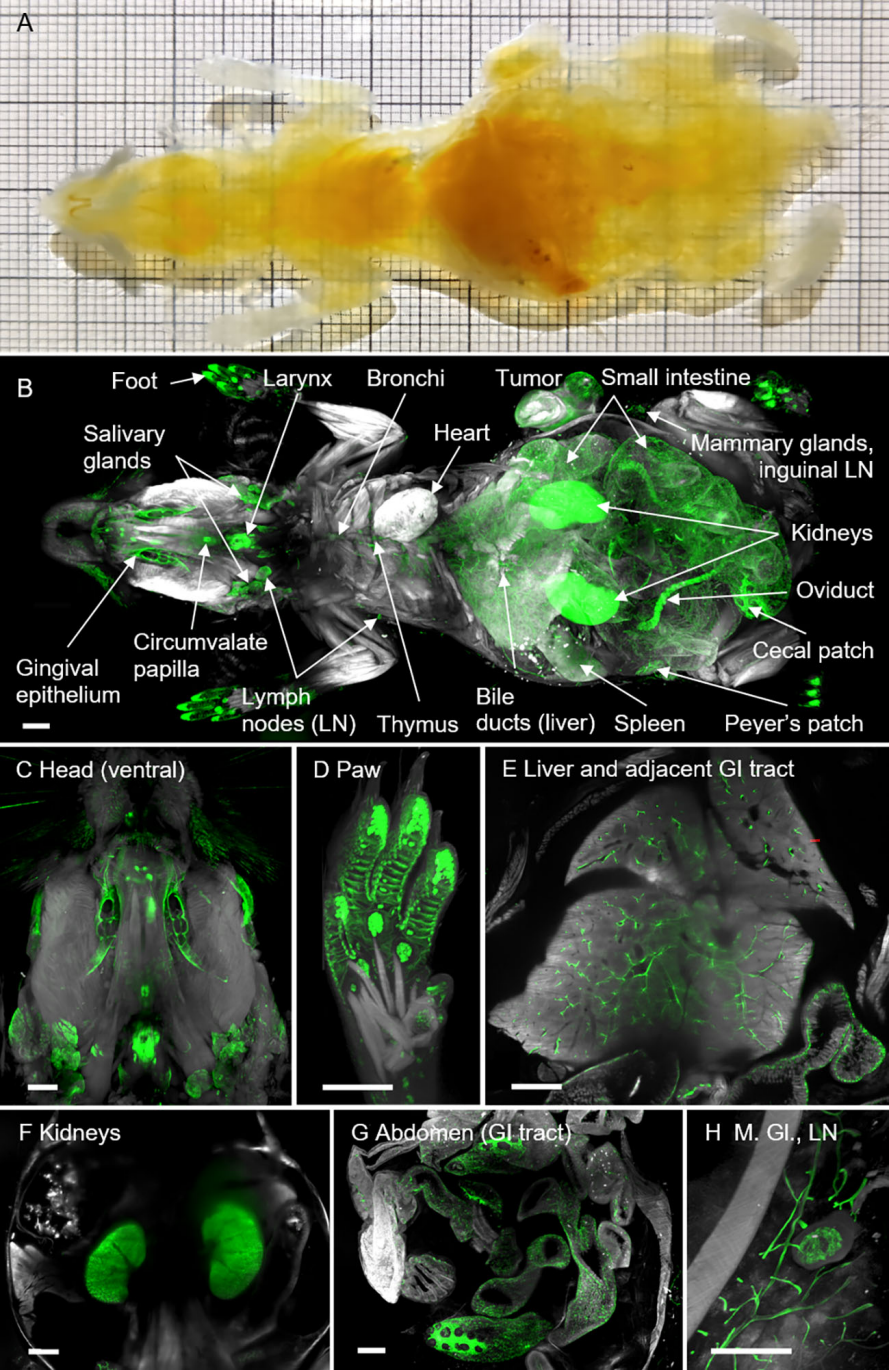
Entire mouse body cleared using the ROCKETS whole-mouse procedure and LSFM imaging reveals holistic biodistribution of anti-EpCAM antibody (G8.8R). **(A)** Mouse body (ventral view) after decalcification, preclearing, dehydration and immersion in BABB shows excellent transparency. Thick squares of the grid = 1 cm. **(B)** LSFM rendering of the tissue’s autofluorescence (grey) and anti-EpCAM staining (G8.8R in green) as overlay enabled quick localization of antibody disposition and identification of positive tissues. **(C-H)** LSFM renderings (ventral views) of EpCAM^+^ tissues (G8.8R in green) *in situ*. M. Gl. = Mammary glands, LN = Lymph node. Scale bars = 2 mm.

Next, we asked whether we could also apply the ROCKETS procedure for whole-body LSFM. In this case, we reasoned to first perfuse mice to avoid autolytic processes and to ensure rapid tissue fixation. Therefore, in contrast to the perfusion-free whole-organ clearing protocol, we perfused whole mice with NBF to ensure timely and thorough fixation, followed by 25% ethylenediaminetetraacetic acid (EDTA) to elute light-absorbing calcified minerals from bones, similar to previous reports (86–88). Subsequently we removed the skin and cleaned the GIT from chyme and feces *in situ* before mice were incubated in the preclearing cocktail for 10-14 days with three exchanges, using a sealable container to prevent excessive evaporation. After washing off the preclearing reagent, we dehydrated and delipidated the mice before RI matching with BABB as described in the next paragraph. The procedure resulted in excellent transparency of entire mouse bodies (**Figure 3A**) and all inner organs could be easily identified in LSFM scans (**Figure 3B**).

### Dehydration, delipidation and RI matching

For dehydration of individual organs (with or without preclearing) we used a tissue processor, which automatically executed dehydration within 4.5 h enhanced by negative pressure (vacuum) and as described previously (48). Brains and whole mice required additional delipidation and, therefore, we manually dehydrated these tissues by adapting the previously published iDISCO+ protocol (89) using methanol (MeOH) and dichloromethane (DCM) (74, 90). Importantly, we omitted the previously described bleaching step with H_2_O_2_ of the original iDISCO+ protocol to avoid rapid quenching of fluorophores induced by oxidative treatments. Irrespective of the applied ROCKETS modules, all specimens were finally immersed in BABB for RI matching and imaging.

### Biodistribution of an anti-EpCAM antibody (G8.8R)

The cell surface glycoprotein EpCAM is highly expressed on a variety of epithelial cancers but also in healthy tissues, successful therapeutic targeting relies on balancing on- and off-tumor effects. To map EpCAM expression throughout the whole organism, we employed our newly established ROCKETS procedure to investigate the biodistribution of the monoclonal anti-EpCAM IgG2a antibody (clone G8.8R, conjugated with AlexaFluor750^®^) after i.v. application into the tail vein of wild-type C57BL/6 mice bearing a subcutaneous ectopic tumor (EpCAM-expressing pancreatic cancer cell line KPC-4662). Upon analysis of the biodistribution, we scored the detected binding levels based on fluorescence intensity levels (**Suppl. Tab. S2**). To account for inherent signal contribution of the autofluorescence, we always scanned negative controls of the same tissue (without antibody) that were equally processed according to the ROCKETS protocol. First, we created LSFM-based 3D renderings of entire mice and mapped EpCAM-(G8.8R-)positive tissues throughout the body (**Figures 3B–H**). Hereby, we could determine individual EpCAM^+^ organs and structures: oral cavity (gingival epithelium) and tongue (gustatory papillae), larynx, thymus, salivary glands, trachea, thymus, bronchi and bronchioles, pancreas, liver (bile canaliculi and gall bladder), GIT (stomach, small intestine, cecum, colon and rectum), kidneys and urinary tract, female reproductive organs (oviducts), mammary glands, foot pads (sweat glands), hair follicles, brain ventricles (choroid plexus) and tumor.

**Figure 4:**
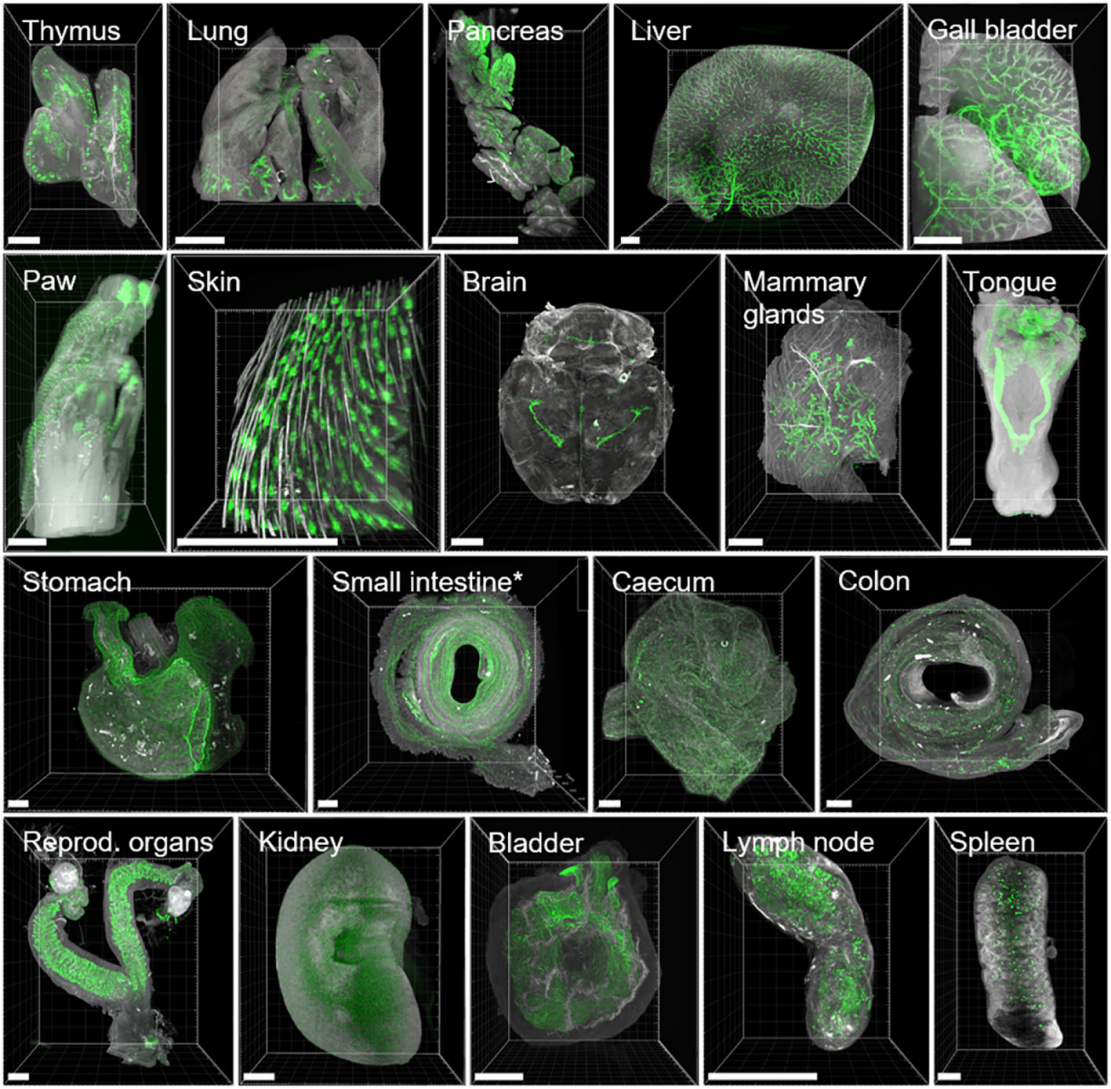
3D renderings of LSFM images show highly heterogeneous binding of anti-EpCAM antibody (G8.8R) between and within organs. EpCAM stainings (G8.8R in green) and tissue anatomy revealed by tissue autofluorescence (grey) in maximum intensity projections (MIPs) of selected positive tissues. EpCAM binding was detected at (but not limited to) previously published sites of EpCAM expression (91). * Only the first of three segments of the small intestine depicted (corresponding to duodenum and proximal jejunum). Scale bars = 1000 μm.

Based on our findings in intact mice, we processed and cleared whole organs individually according to the ROCKETS toolbox to investigate the biodistribution of the EpCAM (G8.8R) antibody at higher magnifications on a cellular level. All tissues that we considered negative in whole-mouse imaging also proved negative upon individual inspection (connective, muscular and nervous tissues, bones). Cuboidal and columnar epithelia clearly stained for EpCAM (G8.8R), as well as lymphoid organs (thymus, LN, Peyer’s patches [PPs] and spleen) (**Figure 4**). Thereby, all known EpCAM^+^ tissues in mice (91–94) were accessed and bound by the anti-EpCAM antibody clone G8.8R *in vivo* within 24 h of circulation. We investigated binding in each organ in detail and could easily determine substructures and individual cells that were positive for the antibody (**Figure 4** and **Suppl. Figure S9**-**22**). For example, we identified individual nephrons in the kidney and determined that binding was restricted to distal convoluted tubules and collecting ducts with distinct binding to intercalated cells and excluded from proximal convoluted tubules and glomeruli (**Suppl. Figure S16**).

**Figure 5:**
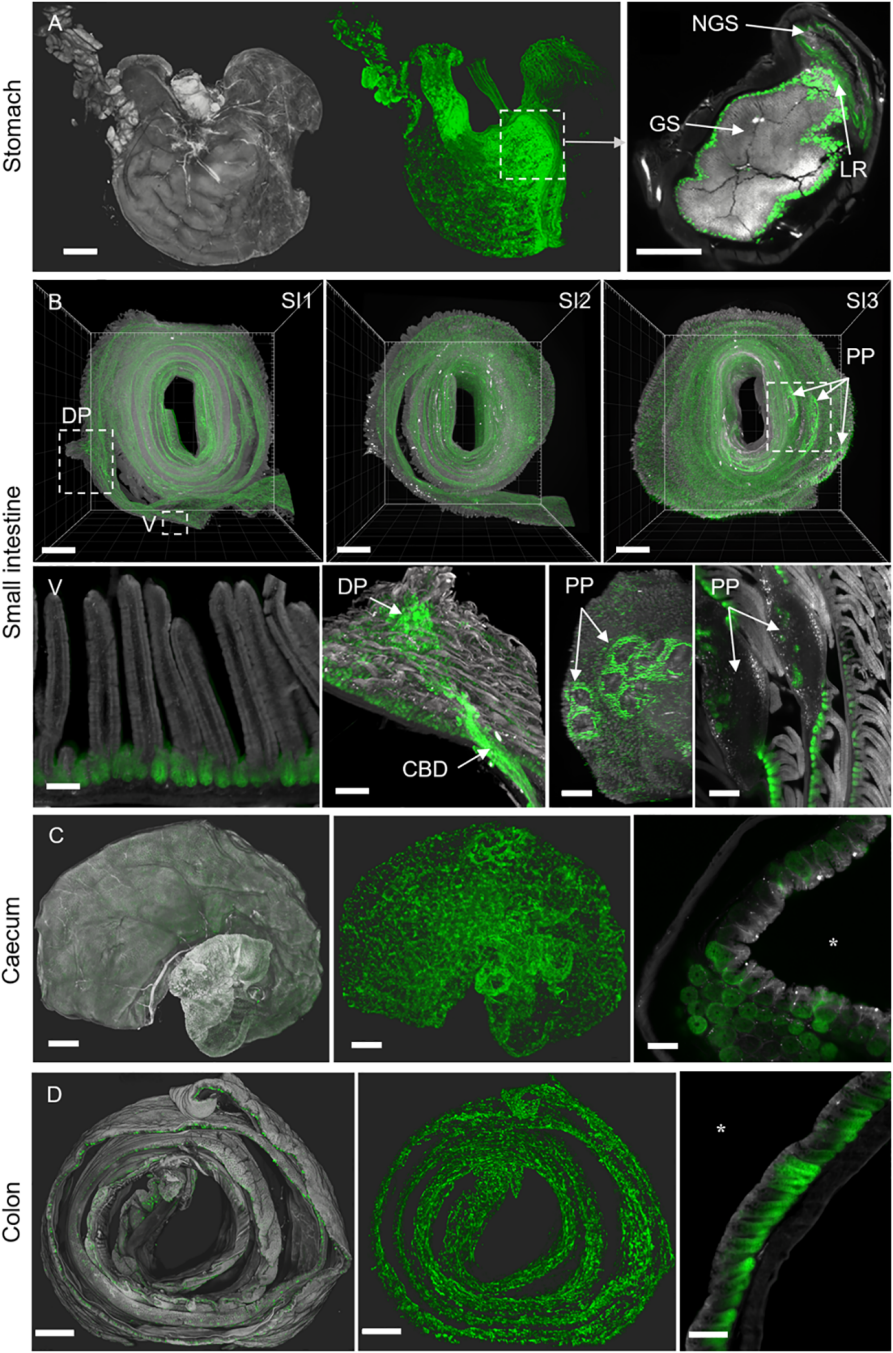
3D-Swiss Rolls present anti-EpCAM staining (G8.8R) in the GIT. Tissue autofluorescence (grey) and anti-EpCAM staining (G8.8R in green). **(A)** Surface renderings of the stomach and associated tissues. Left image depicts tissue anatomy, middle image depicts anti-EpCAM staining (G8.8R) antibody in the same specimen without anatomical context. Right image shows junction of the glandular (GS) and non-glandular stomach (NGS) with increased binding to the glandular mucosa near the limiting ridge (LR) **(B)** All three segments (SI1-3) of the small intestine as 3D-Swiss Rolls (upper row) with indicated duodenal papilla (DP) and several PPs) exposing increased anti-EpCAM binding. Lower row shows higher magnifications of structures as indicated in **(B)** Binding was restricted to basolateral membranes of epithelial cells with pronounced binding to the crypts and decreased or no binding in the villi (V). Anti-EpCAM binding was increased in crypts of the major duodenal papilla, common bile duct (CBD) and near PP compared to overall binding levels in the small intestine. Signals within PP follicles were diffuse and non-membranous, as observed for other lymphoid organs (**Suppl. Figures 6**-**9**). **(C)** Caecum and **(D)** colon show similar patchy binding patterns with decreasing gradients from the proliferative bottom of the crypts towards the luminal surface of the tissues (luminal domain indicated by *asterisk). Scale bars = 1 mm (**A**, **B** SI1-3, **C**, **D**) and 100 μm (all others).

On a subcellular level, binding was restricted to basolateral membranes in all positive epithelia (**Suppl. Figure S9**), which also reflects known EpCAM expression patterns (30). Furthermore, we detected more pronounced binding to proliferative stem cells in crypts of the small intestine than at differentiated enterocytes and goblet cells in the villi, corresponding with described EpCAM downregulation upon differentiation in the GIT (95). Similarly, binding levels gradually decreased from the bottom of the crypts in the cecum and colon towards the luminal surface (**Figures 5C, D** **)**, also corresponding with respectively reported EpCAM expression gradients in rats (96). In lymph nodes, spleen, thymus and inside PP follicles we detected non-polarized membranous and diffuse signals (**Suppl. Figures S10**-**S13**) that may be attributed to low EpCAM expression on T-, B- and dendritic cells in mice (92–94) but may to a certain degree also reflect Fc-dependent binding of the antibody. Within the tumor, EpCAM binding appeared characteristic for carcinomas (30) as non-polarized and highly heterogeneous (**Suppl. Figure S14**).

**Figure 6:**
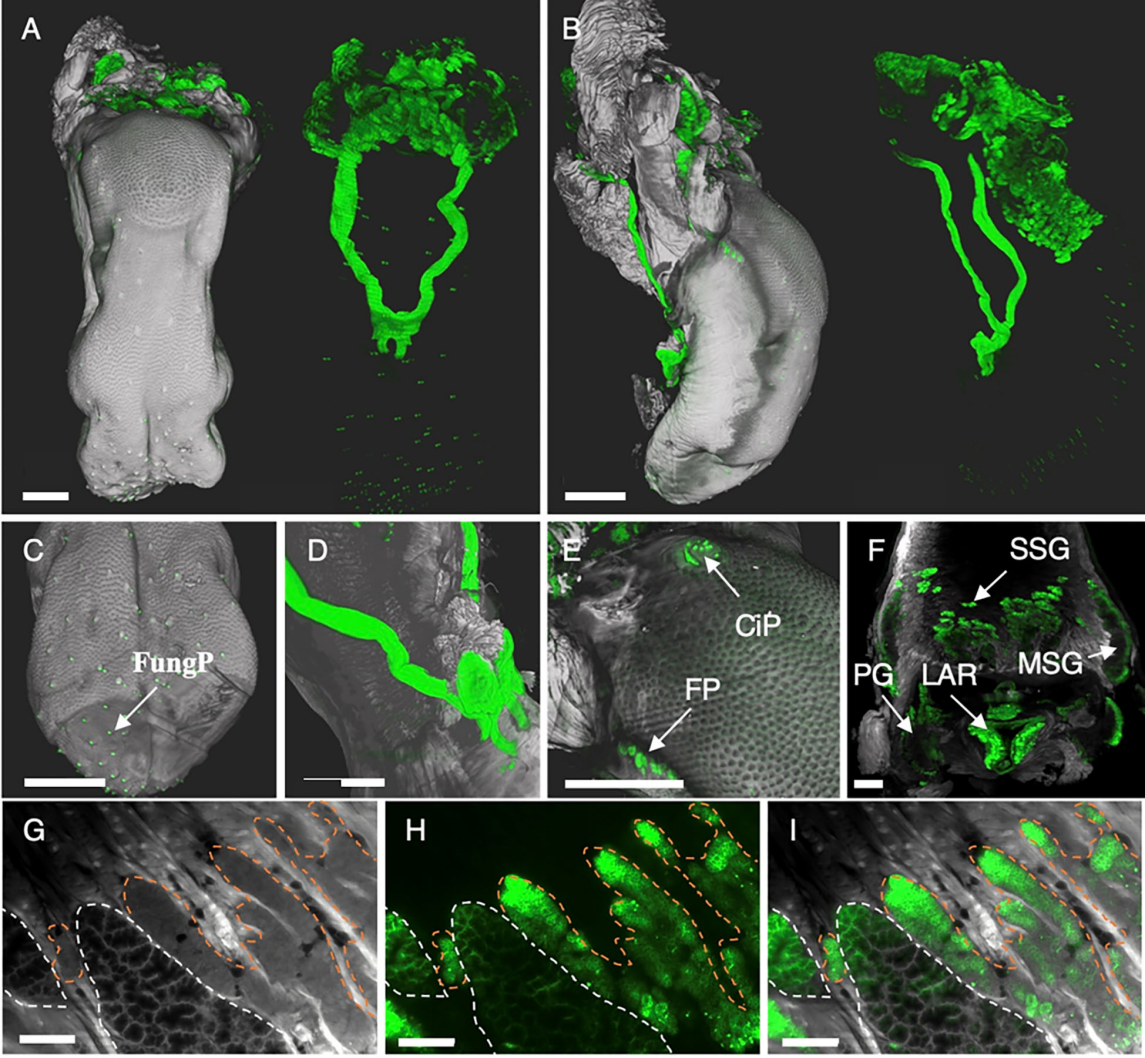
3D renderings and single LSFM images display highly heterogeneous binding of anti-EpCAM antibody G8.8R to the tongue and salivary glands. **(A)** Dorsal and **(B)** lateral view of surface renderings of the tongue and associated tissues. Left images depict renderings of the tissue anatomy (grey) and the bound anti-EpCAM antibody (G8.8R in green) as overlay. Right images depict only the antibody signal (green) without anatomical context. **(C)** Tip of the tongue with positive gustatory fungiform papillae (FungP). **(D)** Positive sublingual excretory ducts at the tongue bottom. **(E)** Circumvallate papilla (CiP) and folate papillae (FP). **(F)** Mucous salivary glands (MSG) and serous salivary glands (SSG), parotid gland (PG) and larynx (LAR). **(G)** Single digital section of the tongue depicting both mucous (white dashed lines) and serous (orange dashed lines) salivary gland anatomy, **(H)** bound G8.8R (green) and **(I)** overlay of both channels. Scale bars = 1 mm **(A–F)** and 150 μm **(G–I)**.

**Figure 7:**
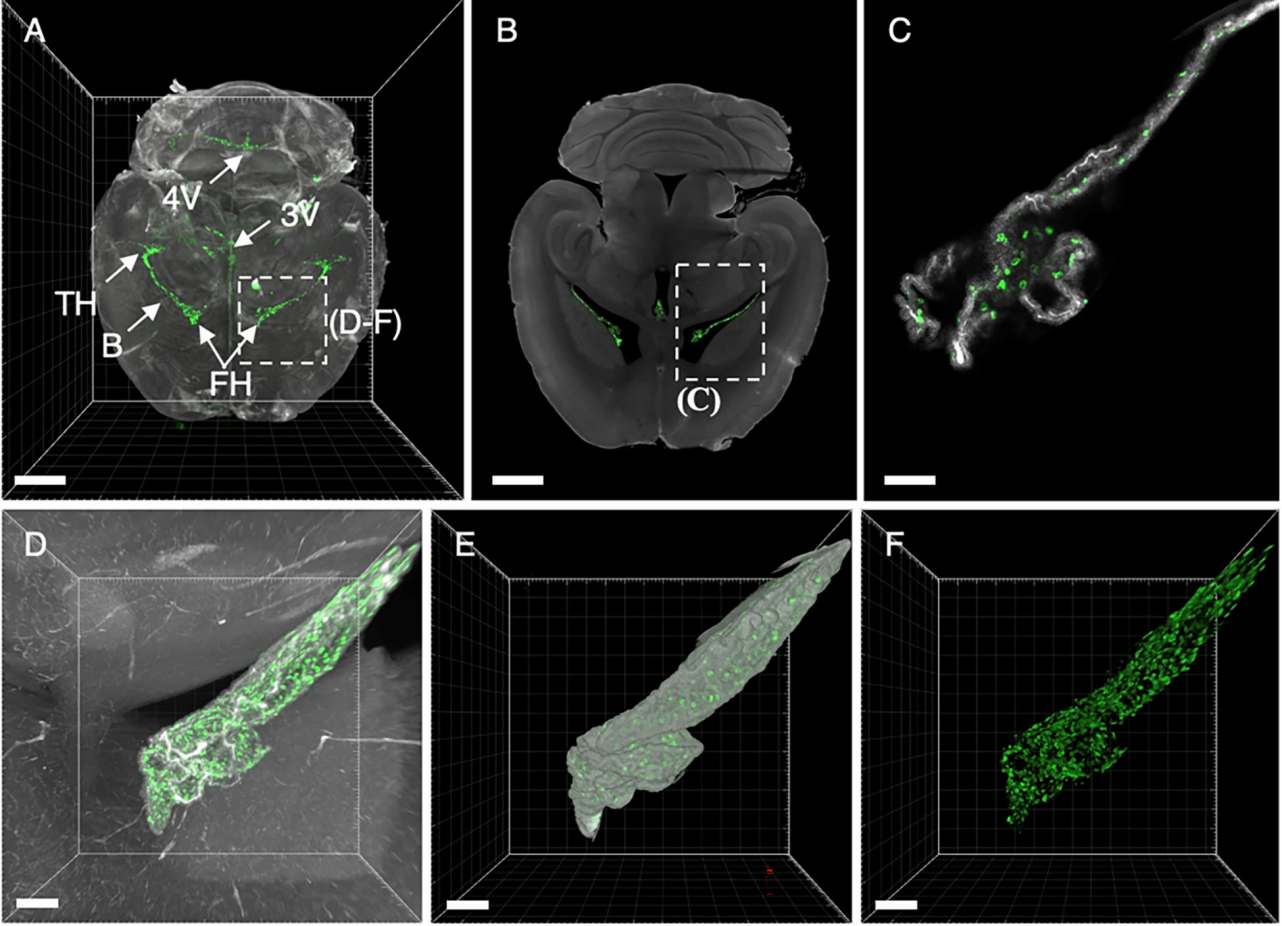
3D renderings and single digital sections reveal EpCAM binding to choroid plexi in the brain. **(A)** Dorsal MIP of the entire brain anatomy derived from the autofluorescence (grey) and binding of the EpCAM-specific antibody G8.8R (green). **(B)** Single LSFM image reveals anti-EpCAM antibody binding to choroid plexi of the temporal horn (TH), frontal horn (FH), 3rd ventricle (3V), 4th ventricle (4V, in A) and body (B, central part). **(C)** Higher magnification image of area indicated in image B displays binding to individual choroid plexus cells. **(D)** Maximum intensity projections (MIP) and **(E, F)** surface renderings of the entire frontal horn choroid plexus with bound anti-EpCAM antibody (G8.8R) extending into the ventricular space as indicated in image **(A)**. Scale bars = 2 mm **(A, B)** and 100 μm **(C–F)**.

Importantly, we further detected anti-EpCAM (G8.8R) stainings in tissues that previously had not been investigated for EpCAM or had even been reported negative. We observed EpCAM expression on the tongue (**Figures 6A, B**) at all types of gustatory papillae (fungiform, circumvallate and foliate, **Figures 6C, E** **)**, which were not addressed in published expression analyses in both mice and humans (28, 97). Mucous salivary gland (MSG) acini were found EpCAM^+^ in some histological studies (98) while others did not detect EpCAM (99) or did not discriminate between mucous and serous salivary glands (SSG) (100). In LSFM scans of lingual salivary glands we observed a heterogeneous binding pattern across the entire gland and generally much lower signals in MSG than in SSG acini (**Figures 6F–I**). Salivary ducts were also found highly EpCAM^+^ (**Figures 6A, B, D**). Similarly, EpCAM expression in choroid plexus (CP) epithelia was not analyzed in investigations of the human brain (100) or had been even described as EpCAM- (98). However, we detected high levels of EpCAM (G8.8R) binding to individual CP cells (**Figure 7**) distinctively delineating the CPs in mouse brains. LSFM scans of 3D-Swiss Rolls allowed us to holistically investigate binding in the entire GIT without the requirement of physical sectioning (**Figure 5**). The stomach showed a highly heterogeneous EpCAM-binding pattern, pronounced at the glandular mucosa directly adjacent to the limiting ridge and in the gastric epithelium throughout the glandular stomach (**Figures 5A, B**). The cornified, stratified squamous epithelium of the forestomach showed very weak signals. In the small intestine the binding patterns and levels were similar throughout the entire length and circumference, restricted to basolateral membranes of epithelial cells (**Figure 5B**). In the small intestine the binding patterns and levels were similar throughout the entire length and circumference, restricted to basolateral membranes of epithelial cells and prominent in the crypts (**Figure 5B**). However, in deviation from gross binding patterns, we detected strong and distinct EpCAM expression at the critical junction where the common bile duct and pancreatic duct drain into the small intestine, namely the major (papilla of Vater) and minor duodenal papillae, but also significantly increased EpCAM levels in the common bile duct (CBD), and in proximity of PPs (**Figure 5B**, lower row). Interestingly, the PP dome epithelium showed low binding levels at the edges and was completely negative at the very center. Within PP follicles EpCAM-binding patterns appeared very similar to lymphoid follicles in LNs, corresponding to their shared immunological function (**Suppl. Figures S9-S11**).

To confirm EpCAM expression at the newly discovered binding sites in mice and humans, we stained-formalin fixed paraffin-embedded (FFPE) tissue sections with different anti-EpCAM antibodies against murine (clone G.8.8) and human (MOC31) EpCAM. Thereby, we independently confirmed EpCAM expression at gustatory papillae and duodenal papillae in both, mice and humans (**Suppl. Figure S8**). Finally, we could also confirm EpCAM expression in the epithelial cells of the choroid plexi in murine FFPE sections.

## Discussion

In this work, we have integrated current knowledge and advanced procedures in tissue clearing to create a substantially simplified, streamlined and versatile sample preparation toolbox for LSFM that we termed ROCKETS. Experimenters may choose a suitable protocol from the ROCKETS toolbox for any mouse organ of interest or entire mouse bodies. The modular manner to apply the appropriate procedure and application should help to efficiently analyze any tissue type of interest or even all mouse organs in a standardized high-throughput mode relevant for basic immunological research but also for thoroughly assessing targets and reagents for novel theragnostic strategies in the preclinical development stage.

For assessing very large and blood-rich organs ROCKETS provides the advantage of efficient clearing with the developed passive two-step approach, which allows to omit transcardial perfusion, which is required for most other published protocols (70). Particularly for large-cohort preclinical animal studies, this simplification is an important element to reduce complexity and effort for tissue clearing. However, our chemical decolorization approach by eluting light-absorbing components does not necessitate perfusion only in terms of optical clearing. Blood remains inside the vessels, which has to be considered when fluorescence-labeled antibodies are applied *via* intravenous injections. In our case, the applied anti-EpCAM antibody was fully cleared from the bloodstream within 24 hours.

For smaller and less vascularized tissues the chemical preclearing step can be entirely omitted to reduce incubation times, waste and expenses. However, clearing of all tissues generally benefits from the preclearing through increased signal-to-noise ratios, which helps to enhance the measurement of even discrete specific signals. Of note, for direct comparison of different tissues within a given experiment, all samples should be treated equally to ensure comparability of fluorescence signal intensities. Apart from sample size and type, the choice of fluorescence probes generally affects clearing requirements. Red or near-infrared emitters may be detectable at high contrast while blue or green emitters can appear blurry because light at respective wavelengths interacts more with biological tissues and is therefore scattered (101). However, imaging of autofluorescence signals revealed that with the presented techniques lower-wavelength emitters may be applied just as well for high-contrast imaging, if e.g. more than one specific signal needs to be detected. Therefore, we recommend applying fluorophores starting from NIR emitters and down to red, yellow, green and blue channels. Consequently, in our study, we chose AlexaFluor750 as the fluorescence dye to label the biodistribution of an EpCAM-specific antibody. Therefore, this reporter was well suited for sensitive detection deep within large organs and even in whole mice.

Histological investigations of the murine GIT are mostly performed using thin slices of tissue fragments or conventional *Swiss rolls* (82–84) or are only focused on particular areas of the intestinal tract (52, 63) and thus, inherently underrepresent its three-dimensional complexity. 3D-Swiss Rolls allowed us to clear and image the small intestine and colon in full length and circumference at cellular resolution without affecting its microanatomy. The holistic imaging revealed that antibody binding was significantly elevated in the vicinity of functionally critical structures like PPs and particularly at the duodenal papillae, which are difficult to locate on histological slices. However, the procedure required quick handling and processing to halt autolytic processes, which take place in gastrointestinal tissue specimens as a result of exposure to gastric acid, bile and digestive enzymes (85). Thus, any study using 3D-Swiss Rolls should be well prepared and the technique practiced in advance, particularly because autolytic damage to the tissues may be mistaken for drug-induced lesions later on.

After dissection and optional preclearing, all organs except brains were dehydrated automatically in a tissue processor without user interference, which further streamlined and simplified the overall process (48). The brain had additionally to be delipidated using MeOH and DCM because of its lipid-rich composition. It should be noted that this difference in dehydration might affect comparability between the brain and other organs in terms of signal intensity. After dehydration, all specimens were cleared using BABB and could therefore be imaged without exchanges of the immersion medium during imaging. In the current study we focused our analyses on mouse tissue specimens. Although not formally proven, we would suggest that the ROCKETS toolbox should be compatible for human specimens as previously we had demonstrated successful BABB-based clearing of human tissue samples (52).

As opposed to tissue processing for single-organ imaging, whole mouse bodies required to ensure timely and thorough fixation as well as decalcification of bones. The remaining clearing process for whole mice was overall simple and fully passive and we could process multiple animals in parallel, limited only by the number of available perfusion pumps. *Ex vivo* LSFM imaging of cleared mouse bodies provided significantly higher resolution than typical *in vivo* imaging methods (102) but also produced very large data sets of several hundred gigabytes of data per animal. Correspondingly, data handling and three-dimensional rendering required significant computing power but then enabled holistic and highly detailed assessment of the biodistribution of the anti-EpCAM antibody for straightforward identification of positive and negative tissues.

Biodistribution mapping showed that all known EpCAM^+^ tissues in mice (91–94) were specifically labeled with the EpCAM antibody clone G8.8R 24 h after *in vivo* administration. Imaging of entire animals also allowed for direct comparison of fluorescence intensity levels to derive semi-quantitative binding scores throughout the body. Accordingly, we observed significant differences in absolute intensity levels between three animals but, importantly, the relative intensity distribution between body regions was equal for all investigated mice.

High-resolution imaging of cleared whole organs confirmed the findings in whole mice and provided more detailed information about binding patterns at a cellular level. Binding in all simple and pseudostratified epithelia was restricted to basolateral membranes, in accordance with known expression patterns (30, 96). The detected signal intensities corroborated published differences in cellular expression levels of EpCAM, which are generally higher on proliferating cells and gradually downregulated upon differentiation (95). This pattern was clearly observed across the small and large intestine, where EpCAM (G8.8R) staining gradually subsided from proliferating zones at the bottom of the crypts towards more differentiated cells of the apical domain. These results underline the high sensitivity of LSFM and great potential for quantitative binding analyses in general.

Importantly, utilizing our ROCKETS procedure, we uncovered in our comprehensive EpCAM-biodistribution studies, tissue sites that were highly EpCAM^+^ but which have either not been sampled in published histological expression analysis or have been explicitly reported as EpCAM^–^ in mice or humans (29, 92, 94, 98, 100). All types of gustatory papillae, which represent clusters of specialized epithelial cells (103), known to express EpCAM in chickens (104), were also detected as EpCAM^+^ in mice.

For salivary glands, some expression analyses did not differentiate between types of salivary glands (100) or defined MSG as EpCAM^–^ (99). In LSFM scans, we detected significant differences in binding levels between lingual SSG (high) and directly adjacent MSG (negative or low). Therefore, we assume MSG as weakly EpCAM^+^ in mice, and attribute seemingly contradictory negative EpCAM stainings of MSGs in histological studies (99, 100) because of under-sampling or masking/loss of epitopes upon cross-linking fixation or processing.

In the brain, we detected no EpCAM expression in nervous tissue, in agreement with reports of human brain samples (100). However, we observed clearly positive CP cells inside all ventricles in contrast to early reports of CP cells and ependymal cells as EpCAM^–^ (98). CPs comprise of simple cuboidal epithelium (105) and express various cell adhesion molecules that are generally associated with EpCAM in all other epithelia (e.g. E-Cadherin) (106). Furthermore, the blood–cerebrospinal fluid (CSF) barrier is implemented by tight junctions between CP cells (107), which are formed under contribution of EpCAM in all other tissues (108). In contrast to nervous tissue of the brain, CP cells can be considered accessible for antibodies because the CP vascularization comprises of fenestrated endothelium, which is generally leaky for macromolecules like the investigated antibody G8.8R (109). Consequently, we could confirm expression of EpCAM on murine FFPE sections using a different antibody. Altogether, our findings strongly indicate that EpCAM is also expressed in human CPs, but final confirmation is still pending.

In summary, LSFM imaging provided unprecedented holistic insight into the biodistribution of an intravenously administered antibody. The great sensitivity and the readily discovered novel binding sites underscore the analytical power and broad spectrum of applications for LSFM imaging in drug discovery. As we discovered duodenal papillae as sites of high EpCAM expression in both mice and humans, our results may have far-reaching implications for preclinical studies and clinical translation of EpCAM-targeted therapeutics as these are the sites of digestive enzyme release from the pancreas. Many clinical studies targeting EpCAM did not reach their primary endpoints in the past due to dose-limiting toxicities like pancreatitis (110, 111) or gastrointestinal-related adverse events (27, 112, 113). In light of our results, even neurotoxicity that in the past had been attributed to vascular leak syndrome or presumed non-specific binding may deserve re-assessment considering the high level of EpCAM^+^ CP cells as important sites for cerebrospinal fluid secretion in the brain (114). Thus, future studies will require to particularly scrutinize immunotherapies whether they also target concomitantly these potential sensitive anatomical locations.

ROCKETS combined with LSFM imaging provides a highly versatile analytical platform for drug discovery. Generally, the described ROCKETS toolbox may be applied for preclinical assessment of any therapeutic compound or other fluorescence-labeled molecules. The methods are simple, make use of cheap reagents and provide sufficient throughput for large-scale studies. Importantly, the procedures are non-destructive for the investigated specimens and can be further processed for histological examination. Therefore, LSFM imaging can be incorporated into existing preclinical analytical workflows. We envision ROCKETS and LSFM not to replace but rather complement gold-standard histological analyses.

However, the technology also carries some inherent limitations to be considered in each study. For example, i.v. administration of labeled antibodies is of limited use for actual expression studies because of potential inaccessibility of target cells *in vivo*. If target expression, or other tissue characteristics such as immune cell infiltration, should be investigated, additional *ex vivo* immunofluorescence stainings can be conducted as demonstrated exemplarily for CD3^+^ and CD19^+^ cells in lymph nodes. The successful lymph node staining suggests that the ROCKETS clearing procedure is generally compatible with multicolor *ex vivo* staining methods of any cell type as described for other tissues elsewhere (66, 75, 115). However, this approach bears limitations on its own because slow antibody diffusion into large tissue specimens still represents a major burden for *ex vivo* staining. We did not investigate if the developed clearing methods preserve fluorescence signals from endogenous reporter proteins but it is likely that the organic solvent-based clearing would diminish fluorescence signals as described previously (70). To circumvent this limitation, experimenters may apply immunofluorescence stainings using antibodies against these fluorescence proteins.

In the future, further development may be focused on even more streamlined processing and automation to further enhance throughput, particularly of whole mice and 3D-Swiss Rolls. Also, more use cases will certainly help to establish ROCKETS as a useful tool for preclinical drug development and thereby boost the integration into established work streams.

## Data availability statement

The raw data supporting the conclusions of this article will be made available by the authors, without undue reservation.

## Ethics statement

The animal study was reviewed and approved by Government of Upper Bavaria (Regierung von Oberbayern, Munich, Germany).

## Author contributions

Conceptualization: JM, AB, TP. Methodology: JM, MD, NO, NA, TP, AR, AB. Investigation: JM, NO, FO, AG, ML, NA, EA-V, HS, TP, AR, AB. Visualization: JM, A-KW, NA, NO, AR. Supervision: JM, AB, TP, SC, FH, PU, MS, MD. Writing—original draft: JM. Writing—review and editing: JM, AB, NO, TP. All authors contributed to the article and approved the submitted version.

## References

[1] Weiner GJ. Building better monoclonal antibody-based therapeutics. Nat Rev Cancer (2015) 15(6):361–70. doi: 10.1038/nrc3930 PMC4491443 25998715

[2] Hansel TT Kropshofer H Singer T Mitchell JA George AJ. The safety and side effects of monoclonal antibodies. Nat Rev Drug Discovery (2010) 9(4):325–38. doi: 10.1038/nrd3003 20305665

[3] Zahavi D Weiner L. Monoclonal antibodies in cancer therapy. Antibodies (Basel Switzerland) (2020) 9(3). doi: 10.3390/antib9030034 PMC7551545 32698317

[4] Goebeler ME Bargou RC. T Cell-engaging therapies - bites and beyond. Nat Rev Clin Oncol (2020) 17(7):418–34. doi: 10.1038/s41571-020-0347-5 32242094

[5] Drago JZ Modi S Chandarlapaty S. Unlocking the potential of antibody-drug conjugates for cancer therapy. Nat Rev Clin Oncol (2021) 18(6):327–44. doi: 10.1038/s41571-021-00470-8 PMC8287784 33558752

[6] Beck A Goetsch L Dumontet C Corvaïa N. Strategies and challenges for the next generation of antibody-drug conjugates. Nat Rev Drug Discovery (2017) 16(5):315–37. doi: 10.1038/nrd.2016.268 28303026

[7] Larson SM Carrasquillo JA Cheung NK Press OW. Radioimmunotherapy of human tumours. Nat Rev Cancer (2015) 15(6):347–60. doi: 10.1038/nrc3925 PMC4798425 25998714

[8] June CH Sadelain M. Chimeric antigen receptor therapy. New Engl J Med (2018) 379(1):64–73. doi: 10.1056/NEJMra1706169 29972754 PMC7433347

[9] Shah NN Fry TJ. Mechanisms of resistance to car T cell therapy. Nat Rev Clin Oncol (2019) 16(6):372–85. doi: 10.1038/s41571-019-0184-6 PMC8214555 30837712

[10] Provasi E Genovese P Lombardo A Magnani Z Liu PQ Reik A. Editing T cell specificity towards leukemia by zinc finger nucleases and lentiviral gene transfer. Nat Med (2012) 18(5):807–15. doi: 10.1038/nm.2700 PMC5019824 22466705

[11] Liddy N Bossi G Adams KJ Lissina A Mahon TM Hassan NJ. Monoclonal tcr-redirected tumor cell killing. Nat Med (2012) 18(6):980–7. doi: 10.1038/nm.2764 22561687

[12] Kim TH Shivdasani RA. Stomach development, stem cells and disease. Development (2016) 143(4):554–65. doi: 10.1242/dev.124891 PMC4760317 26884394

[13] Kantoff PW Higano CS Shore ND Berger ER Small EJ Penson DF. Sipuleucel-T immunotherapy for castration-resistant prostate cancer. New Engl J Med (2010) 363(5):411–22. doi: 10.1056/NEJMoa1001294 20818862

[14] Hilf N Kuttruff-Coqui S Frenzel K Bukur V Stevanović S Gouttefangeas C. Actively personalized vaccination trial for newly diagnosed glioblastoma. Nature (2019) 565(7738):240–5. doi: 10.1038/s41586-018-0810-y 30568303

[15] Lang F Schrörs B Löwer M Türeci Ö Sahin U. Identification of neoantigens for individualized therapeutic cancer vaccines. Nat Rev Drug Discovery (2022) 21(4):261–82. doi: 10.1038/s41573-021-00387-y PMC7612664 35105974

[16] Ayyar BV Arora S O'Kennedy R. Coming-of-Age of antibodies in cancer therapeutics. Trends Pharmacol Sci (2016) 37(12):1009–28. doi: 10.1016/j.tips.2016.09.005 27745709

[17] Sharma P Allison JP. Dissecting the mechanisms of immune checkpoint therapy. Nat Rev Immunol (2020) 20(2):75–6. doi: 10.1038/s41577-020-0275-8 31925406

[18] Sharma P Siddiqui BA Anandhan S Yadav SS Subudhi SK Gao J. The next decade of immune checkpoint therapy. Cancer Discovery (2021) 11(4):838–57. doi: 10.1158/2159-8290.cd-20-1680 33811120

[19] Omar HA Tolba MF. Tackling molecular targets beyond pd-1/Pd-L1: Novel approaches to boost patients' response to cancer immunotherapy. Crit Rev oncology/hematol (2019) 135:21–9. doi: 10.1016/j.critrevonc.2019.01.009 30819443

[20] Wajant H Beilhack A. Targeting regulatory T cells by addressing tumor necrosis factor and its receptors in allogeneic hematopoietic cell transplantation and cancer. Front Immunol (2019) 10:2040. doi: 10.3389/fimmu.2019.02040 31555271 PMC6724557

[21] Medler J Kucka K Melo V Zhang T von Rotenhan S Ulrich J. Cd40- and 41bb-specific antibody fusion proteins with Pdl1 blockade-restricted agonism. Theranostics (2022) 12(4):1486–99. doi: 10.7150/thno.66119 PMC8825603 35198053

[22] Coulie PG Van den Eynde BJ van der Bruggen P Boon T. Tumour antigens recognized by T lymphocytes: At the core of cancer immunotherapy. Nat Rev Cancer (2014) 14(2):135–46. doi: 10.1038/nrc3670 24457417

[23] Syn NL Teng MWL Mok TSK Soo RA. De-Novo and acquired resistance to immune checkpoint targeting. Lancet Oncol (2017) 18(12):e731–e41. doi: 10.1016/s1470-2045(17)30607-1 29208439

[24] June CH Warshauer JT Bluestone JA. Is autoimmunity the achilles' heel of cancer immunotherapy? Nat Med (2017) 23(5):540–7. doi: 10.1038/nm.4321 28475571

[25] Murciano-Goroff YR Warner AB Wolchok JD. The future of cancer immunotherapy: Microenvironment-targeting combinations. Cell Res (2020) 30(6):507–19. doi: 10.1038/s41422-020-0337-2 PMC7264181 32467593

[26] Herlyn M Steplewski Z Herlyn D Koprowski H. (1979). Colorectal carcinoma-specific antigen: Detection by means of monoclonal antibodies, in: Proceedings of the National Academy of Sciences of the United States of America, , Vol. 76. pp. 1438–42. doi: 10.1073/pnas.76.3.1438 PMC383267 286328

[27] Gires O Pan M Schinke H Canis M Baeuerle PA. Expression and function of epithelial cell adhesion molecule epcam: Where are we after 40 years? Cancer Metastasis Rev (2020) 39(3):969–87. doi: 10.1007/s10555-020-09898-3 PMC7497325 32507912

[28] Schnell U Cirulli V Giepmans BN. Epcam: Structure and function in health and disease. Biochim Biophys Acta (2013) 1828(8):1989–2001. doi: 10.1016/j.bbamem.2013.04.018 23618806

[29] Went PT Lugli A Meier S Bundi M Mirlacher M Sauter G. Frequent epcam protein expression in human carcinomas. Hum Pathol (2004) 35(1):122–8. doi: 10.1016/j.humpath.2003.08.026 14745734

[30] van der Gun BT Melchers LJ Ruiters MH de Leij LF McLaughlin PM Rots MG. Epcam in carcinogenesis: The good, the bad or the ugly. Carcinogenesis (2010) 31(11):1913–21. doi: 10.1093/carcin/bgq187 20837599

[31] Silva Lima B Videira MA. Toxicology and biodistribution: The clinical value of animal biodistribution studies. Mol Ther Methods Clin Dev (2018) 8:183–97. doi: 10.1016/j.omtm.2018.01.003 PMC5814363 29541646

[32] Sabdyusheva Litschauer I Becker K Saghafi S Ballke S Bollwein C Foroughipour M. 3d histopathology of human tumours by fast clearing and ultramicroscopy. Sci Rep (2020) 10(1):17619. doi: 10.1038/s41598-020-71737-w 33077794 PMC7572501

[33] Nojima S Susaki EA Yoshida K Takemoto H Tsujimura N Iijima S. Cubic pathology: Three-dimensional imaging for pathological diagnosis. Sci Rep (2017) 7(1):9269. doi: 10.1038/s41598-017-09117-0 28839164 PMC5571108

[34] Wagers AJ Sherwood RI Christensen JL Weissman IL. Little evidence for developmental plasticity of adult hematopoietic stem cells. Sci (New York NY) (2002) 297(5590):2256–9. doi: 10.1126/science.1074807 12215650

[35] Al-Hajj M Wicha MS Benito-Hernandez A Morrison SJ Clarke MF. Prospective identification of tumorigenic breast cancer cells. Proc Natl Acad Sci United States America (2003) 100(7):3983–8. doi: 10.1073/pnas.0530291100 PMC153034 12629218

[36] Reinhardt RL Khoruts A Merica R Zell T Jenkins MK. Visualizing the generation of memory Cd4 T cells in the whole body. Nature (2001) 410(6824):101–5. doi: 10.1038/35065111 11242050

[37] Stelzer EHK Strobl F Chang B-J Preusser F Preibisch S McDole K. Light sheet fluorescence microscopy. Nat Rev Methods Primers (2021) 1(1):73. doi: 10.1038/s43586-021-00069-4

[38] Chatterjee K Pratiwi FW Wu FCM Chen P Chen BC. Recent progress in light sheet microscopy for biological applications. Appl Spectrosc (2018) 72(8):1137–69. doi: 10.1177/0003702818778851 29926744

[39] Belle M Godefroy D Couly G Malone SA Collier F Giacobini P. Tridimensional visualization and analysis of early human development. Cell (2017) 169(1):161–73.e12. doi: 10.1016/j.cell.2017.03.008 28340341

[40] Dodt H-U Leischner U Schierloh A Jährling N Mauch CP Deininger K. Ultramicroscopy: Three-dimensional visualization of neuronal networks in the whole mouse brain. Nat Methods (2007) 4(4):331–6. doi: 10.1038/nmeth1036 17384643

[41] Keller PJ Schmidt AD Santella A Khairy K Bao Z Wittbrodt J. Fast, high-contrast imaging of animal development with scanned light sheet–based structured-illumination microscopy. Nat Methods (2010) 7(8):637–42. doi: 10.1038/nmeth.1476 PMC4418465 20601950

[42] Masselink W Reumann D Murawala P Pasierbek P Taniguchi Y Bonnay F. Broad applicability of a streamlined ethyl cinnamate-based clearing procedure. Development (2019) 146(3):dev166884. doi: 10.1242/dev.166884 30665888 PMC7115989

[43] Tedeschi A Almagro J Renshaw MJ Messal HA Behrens A Petronczki M. Cep55 promotes cytokinesis of neural progenitors but is dispensable for most mammalian cell divisions. Nat Commun (2020) 11(1):1–16. doi: 10.1038/s41467-020-15359-w 32269212 PMC7142149

[44] Ueda HR Erturk A Chung K Gradinaru V Chedotal A Tomancak P. Tissue clearing and its applications in neuroscience. Nat Rev Neurosci (2020) 21(2):61–79. doi: 10.1038/s41583-019-0250-1 31896771 PMC8121164

[45] Hama H Kurokawa H Kawano H Ando R Shimogori T Noda H. Scale: A chemical approach for fluorescence imaging and reconstruction of transparent mouse brain. Nat Neurosci (2011) 14(11):1481–8. doi: 10.1038/nn.2928 21878933

[46] Hama H Hioki H Namiki K Hoshida T Kurokawa H Ishidate F. Scales: An optical clearing palette for biological imaging. Nat Neurosci (2015) 18(10):1518–29. doi: 10.1038/nn.4107 26368944

[47] Casoni F Malone SA Belle M Luzzati F Collier F Allet C. Development of the neurons controlling fertility in humans: New insights from 3d imaging and transparent fetal brains. Development (2016) 143(21):3969–81. doi: 10.1242/dev.139444 27803058

[48] Dobosz M Ntziachristos V Scheuer W Strobel S. Multispectral fluorescence ultramicroscopy: Three-dimensional visualization and automatic quantification of tumor morphology, drug penetration, and antiangiogenic treatment response. Neoplasia (2014) 16(1):1–13. doi: 10.1593/neo.131848 24563615 PMC3924547

[49] Feuchtinger A Walch A Dobosz M. Deep tissue imaging: A review from a preclinical cancer research perspective. Histochem Cell Biol (2016) 146(6):781–806. doi: 10.1007/s00418-016-1495-7 27704211

[50] Schwinn S Mokhtari Z Thusek S Schneider T Sirén A-L Tiemeyer N. Cytotoxic effects and tolerability of gemcitabine and axitinib in a xenograft model for c-myc amplified medulloblastoma. Sci Rep (2021) 11(1):14062. doi: 10.1038/s41598-021-93586-x 34234256 PMC8263612

[51] Brandl A Solimando AG Mokhtari Z Tabares P Medler J Manz H. Junctional adhesion molecule c expression specifies a Cd138low/Neg multiple myeloma cell population in mice and humans. Blood Adv (2022) 6(7):2195–206. doi: 10.1182/bloodadvances.2021004354 PMC9006287 34861679

[52] Brede C Friedrich M Jordan-Garrote AL Riedel SS Bauerlein CA Heinze KG. Mapping immune processes in intact tissues at cellular resolution. J Clin Invest (2012) 122(12):4439–46. doi: 10.1172/JCI65100 PMC3533559 23143304

[53] Amich J Mokhtari Z Strobel M Vialetto E Sheta D Yu Y. Three-dimensional light sheet fluorescence microscopy of lungs to dissect local host immune-aspergillus fumigatus interactions. mBio (2020) 11(1):e02752–19. doi: 10.1128/mBio.02752-19 PMC7002341 32019790

[54] Cordes S Mokhtari Z Bartosova M Mertlitz S Riesner K Shi Y. Endothelial damage and dysfunction in acute graft-Versus-Host disease. Haematologica (2021) 106(8):2147–60. doi: 10.3324/haematol.2020.253716 PMC8327719 32675225

[55] Wertheimer T Velardi E Tsai J Cooper K Xiao S Kloss Christopher C. Production of Bmp4 by endothelial cells is crucial for endogenous thymic regeneration. Sci Immunol (2018) 3(19):eaal2736. doi: 10.1126/sciimmunol.aal2736 29330161 PMC5795617

[56] Shaikh H Vargas JG Mokhtari Z Jarick KJ Ulbrich M Mosca JP. Mesenteric lymph node transplantation in mice to study immune responses of the gastrointestinal tract. Front Immunol (2021) 12:689896. doi: 10.3389/fimmu.2021.689896 34381447 PMC8352558

[57] Susaki EA Ueda HR. Whole-body and whole-organ clearing and imaging techniques with single-cell resolution: Toward organism-level systems biology in mammals. Cell Chem Biol (2016) 23(1):137–57. doi: 10.1016/j.chembiol.2015.11.009 26933741

[58] Richardson DS Lichtman JW. Clarifying tissue clearing. Cell (2015) 162(2):246–57. doi: 10.1016/j.cell.2015.06.067 PMC4537058 26186186

[59] Ariel P. A beginner's guide to tissue clearing. Int J Biochem Cell Biol (2017) 84:35–9. doi: 10.1016/j.biocel.2016.12.009 PMC5336404 28082099

[60] Gómez-Gaviro MV Sanderson D Ripoll J Desco M. Biomedical applications of tissue clearing and three-dimensional imaging in health and disease. iScience (2020) 23(8):101432. doi: 10.1016/j.isci.2020.101432 32805648 PMC7452225

[61] Costantini I Cicchi R Silvestri L Vanzi F Pavone FS. In-vivo and ex-vivo optical clearing methods for biological tissues: Review. BioMed Opt Express (2019) 10(10):5251–67. doi: 10.1364/BOE.10.005251 PMC6788593 31646045

[62] Liu CY Polk DB. Cellular maps of gastrointestinal organs: Getting the most from tissue clearing. Am J Physiol Gastrointest Liver Physiol (2020) 319(1):G1–G10. doi: 10.1152/ajpgi.00075.2020 32421359 PMC7468759

[63] Hülsdünker J Ottmüller KJ Neeff HP Koyama M Gao Z Thomas OS. Neutrophils provide cellular communication between ileum and mesenteric lymph nodes at graft-Versus-Host disease onset. Blood (2018) 131(16):1858–69. doi: 10.1182/blood-2017-10-812891 PMC5909763 29463561

[64] Fu YY Lin CW Enikolopov G Sibley E Chiang AS Tang SC. Microtome-free 3-dimensional confocal imaging method for visualization of mouse intestine with subcellular-level resolution. Gastroenterology (2009) 137(2):453–65. doi: 10.1053/j.gastro.2009.05.008 PMC2894712 19447107

[65] Bernier-Latmani J Petrova TV. High-resolution 3d analysis of mouse small-intestinal stroma. Nat Protoc (2016) 11(9):1617–29. doi: 10.1038/nprot.2016.092 27560169

[66] Almagro J Messal HA Zaw Thin M van Rheenen J Behrens A. Tissue clearing to examine tumour complexity in three dimensions. Nat Rev Cancer (2021) 21(11):718–30. doi: 10.1038/s41568-021-00382-w 34331034

[67] Rogers AB Cormier KS Fox JG. Thiol-reactive compounds prevent nonspecific antibody binding in immunohistochemistry. Lab Invest (2006) 86(5):526–33. doi: 10.1038/labinvest.3700407 16534499

[68] Silvestri L Costantini I Sacconi L Pavone FS. Clearing of fixed tissue: A review from a microscopist's perspective. J Biomed optics (2016) 21(8):81205. doi: 10.1117/1.jbo.21.8.081205 27020691

[69] Tainaka K Kuno A Kubota SI Murakami T Ueda HR. Chemical principles in tissue clearing and staining protocols for whole-body cell profiling. Annu Rev Cell Dev Biol (2016) 32:713–41. doi: 10.1146/annurev-cellbio-111315-125001 27298088

[70] Weiss KR Voigt FF Shepherd DP Huisken J. Tutorial: Practical considerations for tissue clearing and imaging. Nat Protoc (2021) 16(6):2732–48. doi: 10.1038/s41596-021-00502-8 PMC10542857 34021294

[71] Susaki EA Tainaka K Perrin D Kishino F Tawara T Watanabe TM. Whole-brain imaging with single-cell resolution using chemical cocktails and computational analysis. Cell (2014) 157(3):726–39. doi: 10.1016/j.cell.2014.03.042 24746791

[72] Spalteholz W. *Über das durchsichtigmachen Von menschlichen und tierischen präparaten und seine theoretischen bedingungen, nebst anhang: Über knochenfärbung*: S. Hirzel (1914).

[73] Cai R Pan C Ghasemigharagoz A Todorov MI Förstera B Zhao S. Panoptic imaging of transparent mice reveals whole-body neuronal projections and skull-meninges connections. Nat Neurosci (2019) 22(2):317–27. doi: 10.1038/s41593-018-0301-3 PMC6494982 30598527

[74] Erturk A Becker K Jahrling N Mauch CP Hojer CD Egen JG. Three-dimensional imaging of solvent-cleared organs using 3disco. Nat Protoc (2012) 7(11):1983–95. doi: 10.1038/nprot.2012.119 23060243

[75] Renier N Wu Z Simon DJ Yang J Ariel P Tessier-Lavigne M. Idisco: A simple, rapid method to immunolabel Large tissue samples for volume imaging. Cell (2014) 159(4):896–910. doi: 10.1016/j.cell.2014.10.010 25417164

[76] Werner M Chott A Fabiano A Battifora H. Effect of formalin tissue fixation and processing on immunohistochemistry. Am J Surg Pathol (2000) 24(7):1016–9. doi: 10.1097/00000478-200007000-00014 10895825

[77] Au - Gage GJ Au - Kipke DR Au - Shain W. Whole animal perfusion fixation for rodents. JoVE (2012) 65):e3564. doi: 10.3791/3564 PMC3476408 22871843

[78] Williams AC Barry BW. Penetration enhancers. Advanced Drug Delivery Rev (2004) 56(5):603–18. doi: 10.1016/j.addr.2003.10.025 15019749

[79] Weissman I. Genetic and histochemical studies on mouse spleen black spots. Nature (1967) 215(5098):315–. doi: 10.1038/215315a0 6059528

[80] Mowat AM Agace WW. Regional specialization within the intestinal immune system. Nat Rev Immunol (2014) 14(10):667–85. doi: 10.1038/nri3738 25234148

[81] Houston SA Cerovic V Thomson C Brewer J Mowat AM Milling S. The lymph nodes draining the small intestine and colon are anatomically separate and immunologically distinct. Mucosal Immunol (2016) 9(2):468–78. doi: 10.1038/mi.2015.77 26329428

[82] Bialkowska AB Ghaleb AM Nandan MO Yang VW. Improved Swiss-rolling technique for intestinal tissue preparation for immunohistochemical and immunofluorescent analyses. J Vis Exp (2016) 113). doi: 10.3791/54161 PMC4993444 27501188

[83] Moolenbeek C Ruitenberg EJ. The ‘Swiss roll’: A simple technique for histological studies of the rodent intestine. Lab Anim (1981) 15(1):57–60. doi: 10.1258/002367781780958577 7022018

[84] Pereira e Silva A Lourenço AL Marmello BO Bitteti M Teixeira GAPB. Comparison of two techniques for a comprehensive gut histopathological analysis: Swiss roll versus intestine strips. Exp Mol Pathol (2019) 111:104302. doi: 10.1016/j.yexmp.2019.104302 31465765

[85] Williams JM Duckworth CA Vowell K Burkitt MD Pritchard DM. Intestinal preparation techniques for histological analysis in the mouse. Curr Protoc Mouse Biol (2016) 6(2):148–68. doi: 10.1002/cpmo.2 27248432

[86] Jing D Zhang S Luo W Gao X Men Y Ma C. Tissue clearing of both hard and soft tissue organs with the pegasos method. Cell Res (2018) 28(8):803–18. doi: 10.1038/s41422-018-0049-z PMC6082844 29844583

[87] Tainaka K Murakami TC Susaki EA Shimizu C Saito R Takahashi K. Chemical landscape for tissue clearing based on hydrophilic reagents. Cell Rep (2018) 24(8):2196–210.e9. doi: 10.1016/j.celrep.2018.07.056 30134179

[88] Treweek JB Chan KY Flytzanis NC Yang B Deverman BE Greenbaum A. Whole-body tissue stabilization and selective extractions *Via* tissue-hydrogel hybrids for high-resolution intact circuit mapping and phenotyping. Nat Protoc (2015) 10(11):1860–96. doi: 10.1038/nprot.2015.122 PMC4917295 26492141

[89] Renier N Adams EL Kirst C Wu Z Azevedo R Kohl J. Mapping of brain activity by automated volume analysis of immediate early genes. Cell (2016) 165(7):1789–802. doi: 10.1016/j.cell.2016.05.007 PMC4912438 27238021

[90] Erturk A Mauch CP Hellal F Forstner F Keck T Becker K. Three-dimensional imaging of the unsectioned adult spinal cord to assess axon regeneration and glial responses after injury. Nat Med (2011) 18(1):166–71. doi: 10.1038/nm.2600 22198277

[91] Trzpis M McLaughlin PM Popa ER Terpstra P van Kooten TG de Leij LM. Epcam homologues exhibit epithelial-specific but different expression patterns in the kidney. Transgenic Res (2008) 17(2):229–38. doi: 10.1007/s11248-007-9141-8 17940847

[92] Borkowski TA Nelson AJ Farr AG Udey MC. Expression of Gp40, the murine homologue of human epithelial cell adhesion molecule (Ep-cam), by murine dendritic cells. Eur J Immunol (1996) 26(1):110–4. doi: 10.1002/eji.1830260117 8566052

[93] Bergsagel PL Victor-Kobrin C Timblin CR Trepel J Kuehl WM. A murine cdna encodes a pan-epithelial glycoprotein that is also expressed on plasma cells. J Immunol (1992) 148(2):590–6. doi: 10.4049/jimmunol.148.2.590 1729376

[94] Nelson AJ Dunn RJ Peach R Aruffo A Farr AG. The murine homolog of human ep-cam, a homotypic adhesion molecule, is expressed by thymocytes and thymic epithelial cells. Eur J Immunol (1996) 26(2):401–8. doi: 10.1002/eji.1830260220 8617310

[95] Huang L Yang Y Yang F Liu S Zhu Z Lei Z. Functions of epcam in physiological processes and diseases (Review). Int J Mol Med (2018) 42(4):1771–85. doi: 10.3892/ijmm.2018.3764 PMC6108866 30015855

[96] Schiechl H Dohr G. Immunohistochemical studies of the distribution of a basolateral-membrane protein in intestinal epithelial cells (Gz1-Ag) in rats using monoclonal antibodies. Histochemistry (1987) 87(5):491–8. doi: 10.1007/BF00496823 3323146

[97] Trzpis M Popa ER McLaughlin PMJ van Goor H Timmer A Bosman GW. Spatial and temporal expression patterns of the epithelial cell adhesion molecule (Epcam/Egp-2) in developing and adult kidneys. Nephron Exp Nephrol (2007) 107(4):e119–e31. doi: 10.1159/000111039 18025791

[98] Momburg F Moldenhauer G Hämmerling GJ Möller P. Immunohistochemical study of the expression of a Mr 34,000 human epithelium-specific surface glycoprotein in normal and malignant tissues. Cancer Res (1987) 47(11):2883–91. 3552208

[99] Kamal NM Salem HM Dahmoush HM. Immunohistochemical expression of epithelial cell adhesion molecule (Epcam) in mucoepidermoid carcinoma compared to normal salivary gland tissues. Arch Oral Biol (2017) 79:87–94. doi: 10.1016/j.archoralbio.2017.03.014 28347886

[100] Balzar M Winter MJ de Boer CJ Litvinov SV. The biology of the 17–1a antigen (Ep-cam). J Mol Med (1999) 77(10):699–712. doi: 10.1007/s001099900038 10606205

[101] Tuchin VV. Tissue optics and photonics: Light-tissue interaction. 2015 (2015) 1(2):37. doi: 10.18287/JBPE-2015-1-2-98

[102] Arms L Smith DW Flynn J Palmer W Martin A Woldu A. Advantages and limitations of current techniques for analyzing the biodistribution of nanoparticles. Front Pharmacol (2018) 9:802(802). doi: 10.3389/fphar.2018.00802 30154715 PMC6102329

[103] Witt M Kasper M. Distribution of cytokeratin filaments and vimentin in developing human taste buds. Anat Embryol (1999) 199(4):291–9. doi: 10.1007/s004290050229 10195304

[104] Venkatesan N Rajapaksha P Payne J Goodfellow F Wang Z Kawabata F. Distribution of A-gustducin and vimentin in premature and mature taste buds in chickens. Biochem Biophys Res Commun (2016) 479(2):305–11. doi: 10.1016/j.bbrc.2016.09.064 PMC5048587 27639649

[105] Redzic ZB Segal MB. The structure of the choroid plexus and the physiology of the choroid plexus epithelium. Advanced Drug Delivery Rev (2004) 56(12):1695–716. doi: 10.1016/j.addr.2004.07.005 15381330

[106] Szmydynger-Chodobska J Pascale CL Pfeffer AN Coulter C Chodobski A. Expression of junctional proteins in choroid plexus epithelial cell lines: A comparative study. Cerebrospinal Fluid Res (2007) 4:11. doi: 10.1186/1743-8454-4-11 18162136 PMC2241822

[107] Liddelow SA. Development of the choroid plexus and blood-csf barrier. Front Neurosci (2015) 9:32(32). doi: 10.3389/fnins.2015.00032 25784848 PMC4347429

[108] Lei Z Maeda T Tamura A Nakamura T Yamazaki Y Shiratori H. Epcam contributes to formation of functional tight junction in the intestinal epithelium by recruiting claudin proteins. Dev Biol (2012) 371(2):136–45. doi: 10.1016/j.ydbio.2012.07.005 22819673

[109] Dermietzel R Krause D. Molecular anatomy of the blood-brain barrier as defined by immunocytochemistry. In: Jeon KW Friedlander M, editors. International review of cytology, vol. 127 . Academic Press (1991). p. 57–109. 10.1016/s0074-7696(08)60692-0 1880006

[110] De Bono JS Tolcher AW Forero A Vanhove GF Takimoto C Bauer RJ. Ing-1, a monoclonal antibody targeting ep-cam in patients with advanced adenocarcinomas. Clin Cancer Res (2004) 10(22):7555–65. doi: 10.1158/1078-0432.CCR-04-0729 15569986

[111] Goel S Bauer RJ Desai K Bulgaru A Iqbal T Strachan BK. Pharmacokinetic and safety study of subcutaneously administered weekly ing-1, a human engineere monoclonal antibody targeting human epcam, in patients with advanced solid tumors. Ann Oncol (2007) 18(10):1704–7. doi: 10.1093/annonc/mdm280 17693421

[112] Kebenko M Goebeler M-E Wolf M Hasenburg A Seggewiss-Bernhardt R Ritter B. A multicenter phase 1 study of solitomab (Mt110, amg 110), a bispecific Epcam/Cd3 T-cell engager (Bite^®^) antibody construct, in patients with refractory solid tumors. OncoImmunology (2018) 7(8):e1450710. doi: 10.1080/2162402X.2018.1450710 30221040 PMC6136859

[113] Macdonald J Henri J Roy K Hays E Bauer M Veedu RN. Epcam immunotherapy versus specific targeted delivery of drugs. Cancers (Basel) (2018) 10(1). doi: 10.3390/cancers10010019 PMC5789369 29329202

[114] Andersson Y Engebraaten O Juell S Aamdal S Brunsvig P Fodstad Ø. Phase I trial of epcam-targeting immunotoxin Moc31pe, alone and in combination with cyclosporin. Br J Cancer (2015) 113(11):1548–55. doi: 10.1038/bjc.2015.380 PMC4705890 26554649

[115] Messal HA Almagro J Zaw Thin M Tedeschi A Ciccarelli A Blackie L. Antigen retrieval and clearing for whole-organ immunofluorescence by flash. Nat Protoc (2021) 16(1):239–62. doi: 10.1038/s41596-020-00414-z 33247285

