# Supplementary material for: ROCKETS – a novel one-for-all toolbox for light sheet microscopy in drug discovery
Source: Front Immunol. 2023 Feb 7;14:1034032. doi: 10.3389/fimmu.2023.1034032 (PMC9945347; doi:10.3389/fimmu.2023.1034032)
Supplement: Supplementary file 1 [file DataSheet_1.docx]

Supplementary Material

ROCKETS - a novel one-for-all toolbox for light sheet microscopy in drug discovery

**Joerg PJ Mueller^1,2*^, Michael Dobosz^2^, Nils O'Brien^2^, Nassri Abdoush^2^, Anna Maria Giusti^3^, Martin Lechmann^3^, Franz Osl^2^, Ann-Katrin Wolf^1,2^, Estibaliz Arellano-Viera^1^, Haroon Shaikh^1^, Markus Sauer^4^, Andreas Rosenwald^5^, Frank Herting^2^, Pablo Umana^3^, Sara Colombetti^3^, Thomas Pöschinger^2†^, Andreas Beilhack^1†*^**

^1^ Interdisciplinary Center for Clinical Research Laboratory (IZKF) Würzburg, Department of Internal Medicine II, Center for Experimental Molecular Medicine, Würzburg University Hospital, Würzburg, Germany

^2^ Roche Pharmaceutical Research and Early Development, Roche Diagnostics GmbH, Nonnenwald 2, Penzberg, Germany

^3^ Roche Pharmaceutical Research and Early Development, Roche Glycart AG, Wagistraße 10, Schlieren, Switzerland

^4^ Department of Biotechnology and Biophysics, Biocenter, University of Würzburg, Würzburg, Germany

^5^ Institute of Pathology, University of Würzburg, Würzburg, Germany

^†^ TP and AB contributed equally to this work.

^*^ Corresponding authors: JPJM or AB


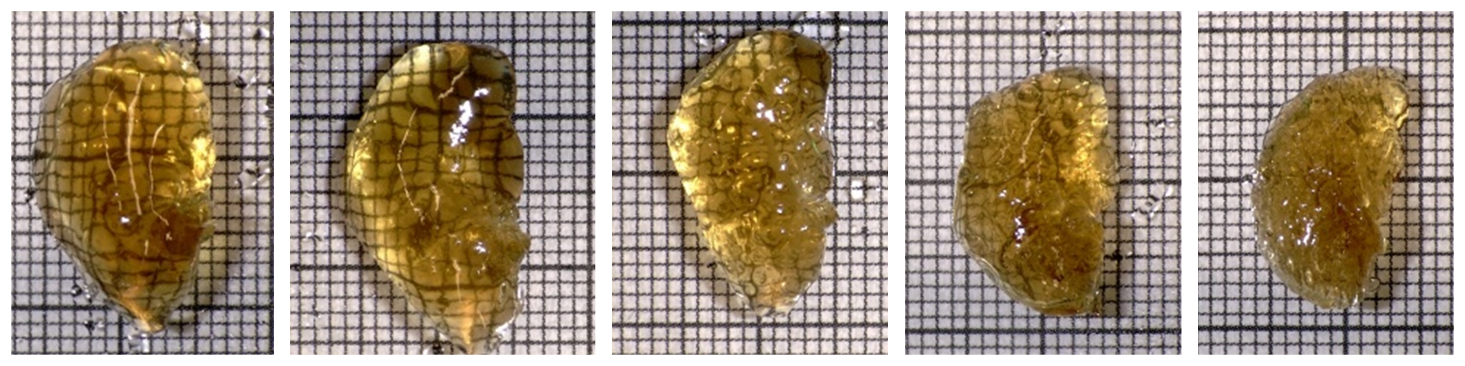
**Supplementary Figure S1. Cleared whole mouse liver lobe specimens after incubation** **with the preclearing reagent with indicated concentrations of urea.** Urea concentrations of 15% (w/v) or higher induced deformation of liver (shown: left lateral lobe) and other tissues that was not reversed upon dehydration and clearing. Thick grid lines = 1 cm.

Saturated solution

No urea

10 % urea

15 % urea

25 % urea

**
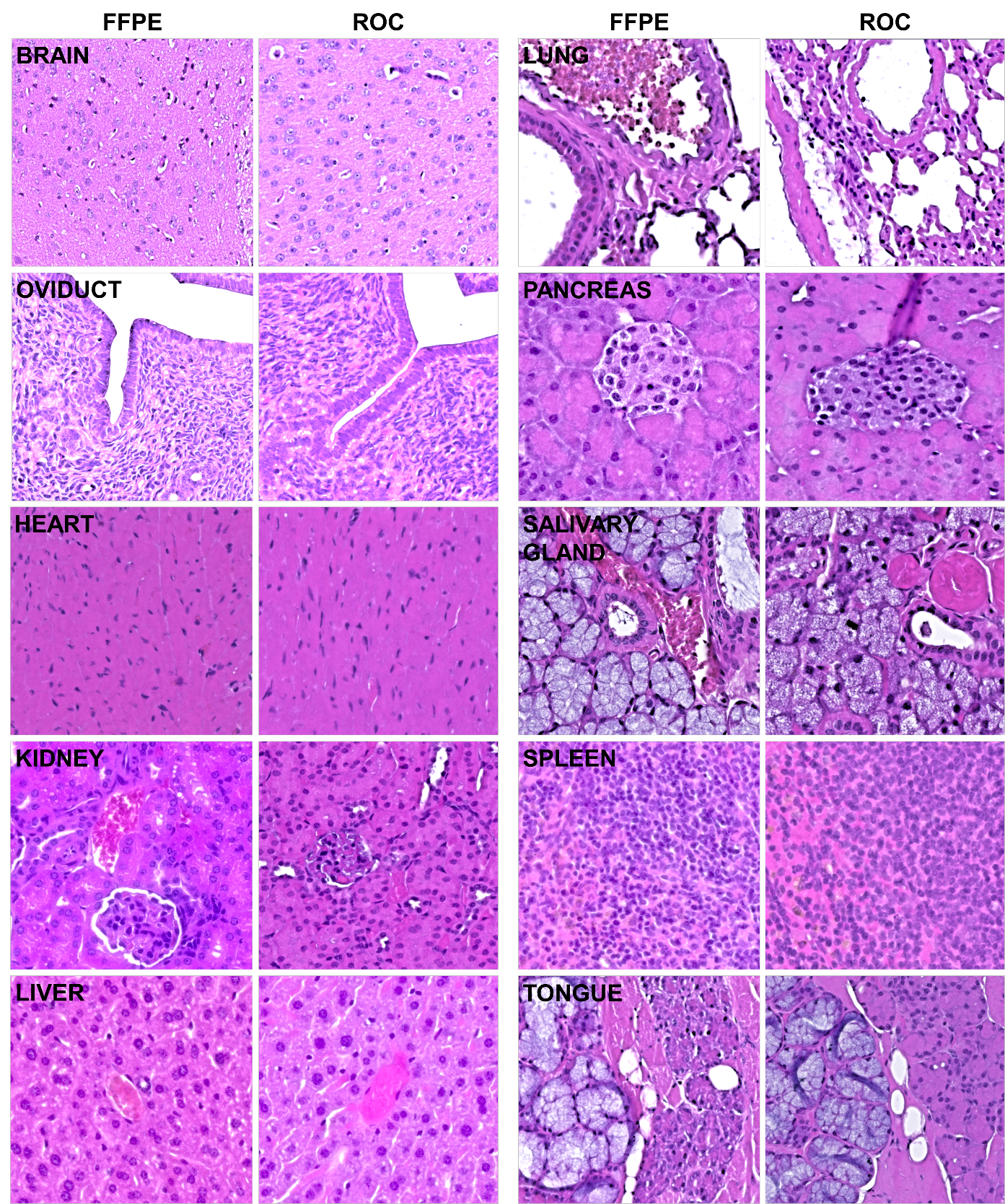

Supplementary Figure S2. Hematoxylin and eosin (H&E) stainings of major mouse organs after standard processing for formalin fixed paraffin embedded tissue sections (FFPE, left columns) or after clearing according to the ROCKETS procedures (ROC, right columns).** Anatomical integrity and H&E stainings were unaffected by the clearing procedures. The preclearing reagent altered erythrocyte staining due to the removal of heme (arrows). Scale bars = 20 µm.

|  | **ROCKETS precleared** | | | | | **Untreated (PBS)** | | | | |
| --- | --- | --- | --- | --- | --- | --- | --- | --- | --- | --- |
| **Channel** | **1** | **2** | **3** | **4** | **5** | **1** | **2** | **3** | **4** | **5** |
| **Ex. λ [nm]** | **470** | **545** | **630** | **685** | **747** | **470** | **545** | **630** | **685** | **747** |
| **Spectrum** | 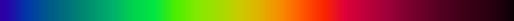 | | | | | 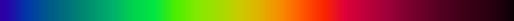 | | | | |
| Kidney | (✔) | ✔ | ✔ | ✔ | ✔ | 🗶 | 🗶 | (✔) | ✔ | ✔ |
| Tongue | ✔ | ✔ | ✔ | ✔ | ✔ | (✔) | ✔ | ✔ | ✔ | ✔ |
| Heart | (✔) | ✔ | ✔ | ✔ | ✔ | 🗶 | 🗶 | 🗶 | ✔ | ✔ |
| Liver | (✔) | ✔ | ✔ | ✔ | ✔ | 🗶 | 🗶 | 🗶 | 🗶 | (✔) |
| Spleen | (✔) | ✔ | ✔ | ✔ | ✔ | 🗶 | 🗶 | (✔) | ✔ | ✔ |
| Stomach | ✔ | ✔ | ✔ | ✔ | ✔ | (✔) | ✔ | ✔ | ✔ | ✔ |
| Sm. int. (SR) | (✔) | ✔ | ✔ | ✔ | ✔ | 🗶 | (✔) | ✔ | ✔ | ✔ |
| L. int. (SR) | ✔ | ✔ | ✔ | ✔ | ✔ | 🗶 | (✔) | ✔ | ✔ | ✔ |
| Caecum | ✔ | ✔ | ✔ | ✔ | ✔ | (✔) | ✔ | ✔ | ✔ | ✔ |
| Fem. repr. | ✔ | ✔ | ✔ | ✔ | ✔ | ✔ | ✔ | ✔ | ✔ | ✔ |
| Lungs | ✔ | ✔ | ✔ | ✔ | ✔ | 🗶 | (✔) | ✔ | ✔ | ✔ |
| Sal. Glands | ✔ | ✔ | ✔ | ✔ | ✔ | 🗶 | 🗶 | ✔ | ✔ | ✔ |
| Pancreas | (✔) | ✔ | ✔ | ✔ | ✔ | (✔) | ✔ | ✔ | ✔ | ✔ |
| Lymph node | ✔ | ✔ | ✔ | ✔ | ✔ | ✔ | ✔ | ✔ | ✔ | ✔ |
| Bladder | ✔ | ✔ | ✔ | ✔ | ✔ | ✔ | ✔ | ✔ | ✔ | ✔ |
| Mam. Glands | ✔ | ✔ | ✔ | ✔ | ✔ | ✔ | ✔ | ✔ | ✔ | ✔ |
| Thymus | ✔ | ✔ | ✔ | ✔ | ✔ | 🗶 | (✔) | ✔ | ✔ | ✔ |

**Supplementary Table S1.** **Channel usability for whole-organ imaging of non-perfused murine specimens with LSFM after ROCKETS preclearing or untreated.** Sm./L. int. = Small/Large intestine; SR = Swiss Roll; Fem. repr. = Female reproductive tract. Channel usability is defined as:

✔ = full-depth high-contrast imaging at homogenous trans-illumination

(✔) = full-depth high-contrast imaging, inhomogeneous trans-illumination with brightness gradient towards center

🗶 = imaging of entire sample not possible using the respective channel (blurry or dark center)


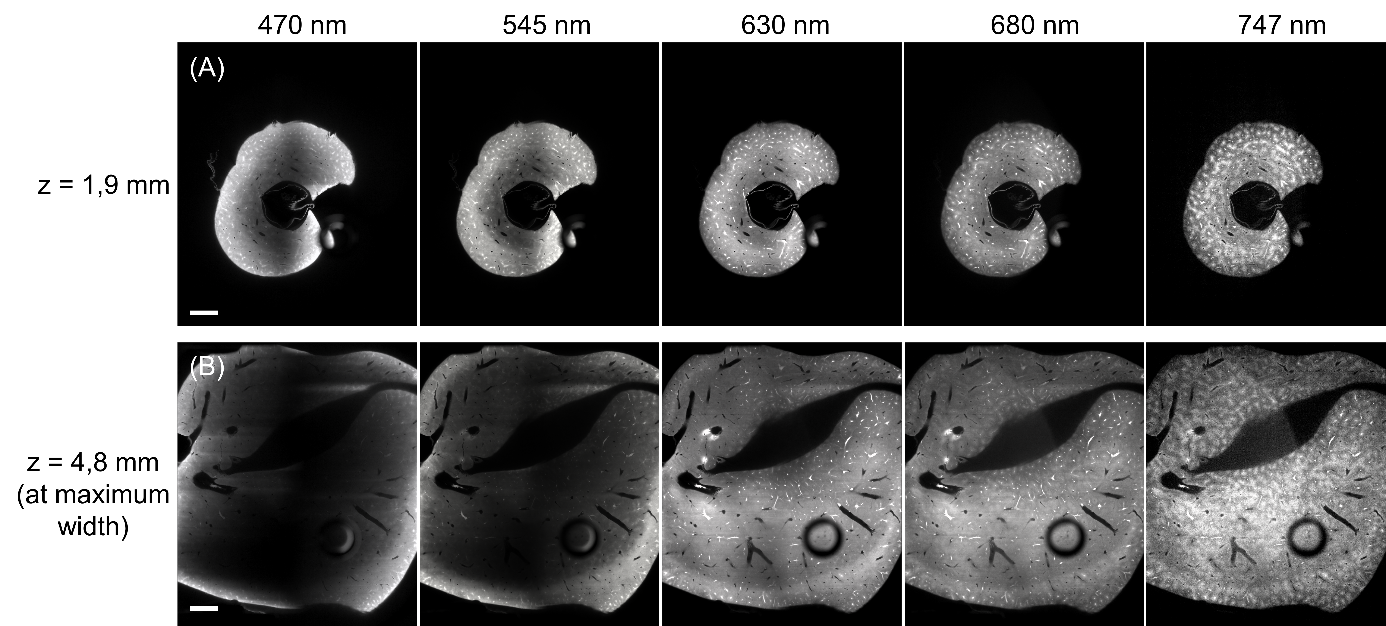


**Suppl. Fig. S3 Imaging quality depends on depth and wavelength.** Among mouse tissues the liver is the optically densest and therefore one of few organs that –despite preclearing– cannot be imaged entirely at every wavelength with LSFM. Homogenous illumination is impeded at shorter wavelengths but can be achieved at longer wavelengths**.**  **(A)** Autofluorescence of a liver specimen imaged closely to the top of the sample (z = 1,9 mm) shows homogenous illumination with good image quality at all available channels (excitation wavelengths are depicted). **(B)** At the deepest point at full width of the same specimen (4,8 mm from the top), channels 1 (470nm) and 2 (545nm) show inhomogeneous illumination due to a longer path that the light must propagate through tissue before it reaches the detector. However, at longer wavelengths homogenous illumination can be achieved (here: optimum at 747nm). Scale bars = 50 µm.

**
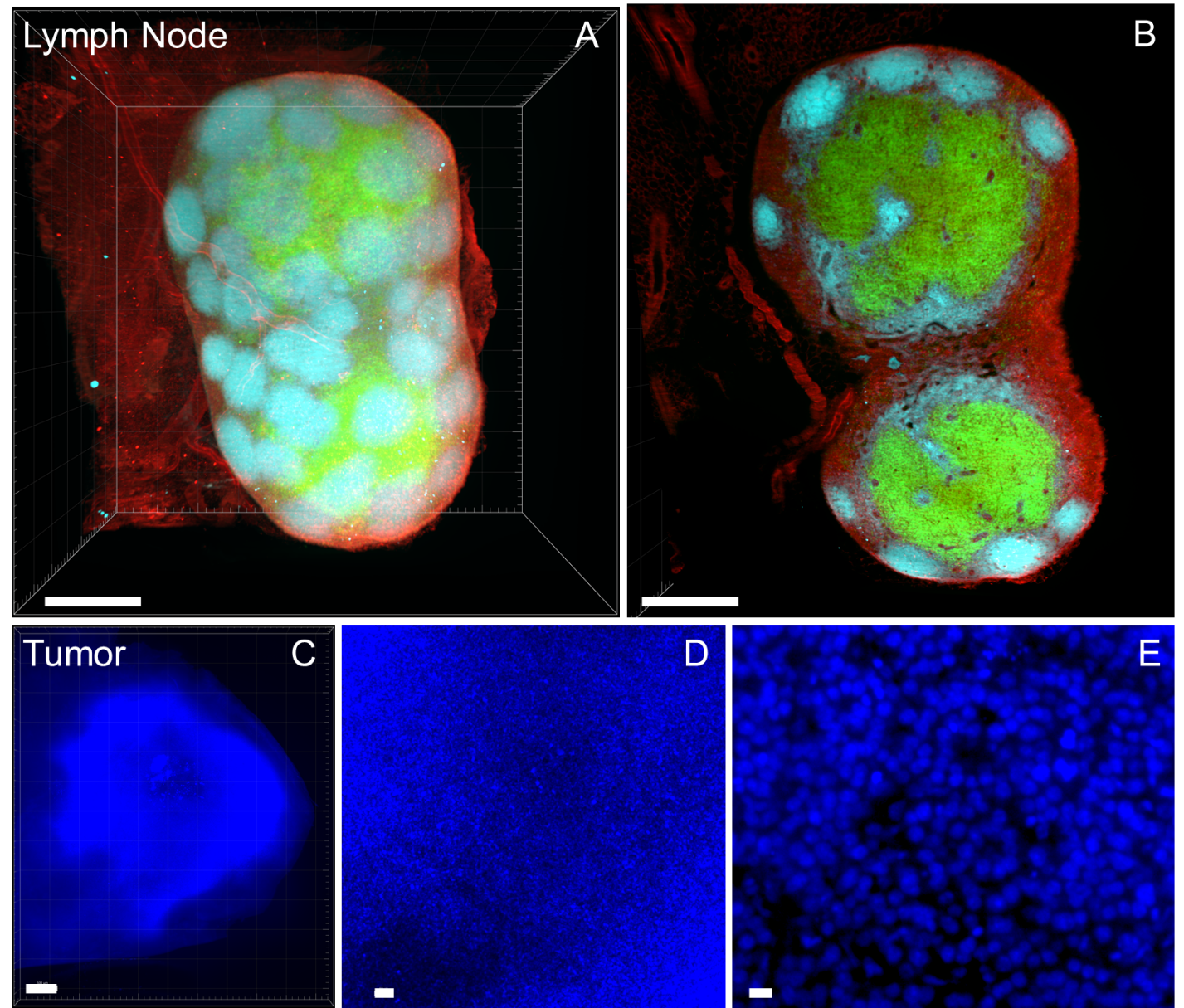
**

**Supplementary Figure S4. LSFM immunofluorescence staining of a lymph node (LN) and nuclear staining of a tumor specimen.** (**A, B**) Upper row shows a whole lymph node, stained with fluorescently labeled antibodies against CD19 (cyan) and CD3 (green), demarking clearly visible B cell follicles and T cell zones in 3D-renderings and single LSFM image. Scale bars = 500 µm. (**C-E**) Tumor specimen stained with propidium iodide for detection of cell nuclei. Higher magnification imaging (**E**) shows that individual nuclei can be distinguished. Scale bars = 50 µm (C, D) and 10 µm (E).

| **Tissue/Structure** | | | **Score** | **Tissue/Structure** | | | **Score** |
| --- | --- | --- | --- | --- | --- | --- | --- |
| **Kidneys and urinary tract** | | |  | **Mammary glands** | | |  |
|  | Glomeruli | |  |  | Ductal epithelium | | +++ |
|  | | Bowman's capsule | - |  | Alveolar epithelium | | - |
|  |  | Capillary tufts | - | **Salivary glands** | | |  |
|  | Proximal convoluted tubules | | - |  | Serous acinar cells | | ++ |
|  | Henle's loops | |  |  | Mucous acinar cells | | + |
|  |  | Descending loop | - |  | Ductal cells | | ++ |
|  |  | Ascending loop | - | **Oral cavity** | | |  |
|  |  | Collecting ducts* | +++ |  | Tongue, squamous epithelium | | - |
|  | Medulla† | | ++ |  | Gustatory papillae (foliate, fungiform and circumvallate) | | ++ |
|  | Pelvis† | | + |  |  |  |  |
|  | Ureter | | + |  | Larynx | | ++ |
|  | Bladder epithelium | | + | **Stomach** | | |  |
|  | Urethra | | + |  | Esophagus tunica mucosa | | + |
| **Lung** | | |  |  | Glandular gastric epithelium§ | | +/++ |
|  | Tracheal mucosa | | + |  | Forestomach squamous epithelium | | - |
|  | Bronchi | | + |  | Limiting ridge epithelium | | + |
|  | Bronchioli | | ++ |  | Pylorus epithelium§ | | +/++ |
|  | Alveolar AT1 cells | | - | **Small intestine**\|\| | | |  |
|  | Alveolar AT2 cells | | + |  | Villi, epithelial cells | | + |
| **Pancreas** | | |  |  | Crypts, epithelial cells | | ++ |
|  |  | Acinar cells | + |  | Duodenal papillae, cmn. bile duct | | +++ |
|  |  | Duct cells | + |  | Peyer's patch, adjacent epithelium | | +++ |
|  |  | Islet cells | - |  | Peyer's patch, follicles¶ | | + |
| **Liver** | | |  | **Large intestine (caecum, colon, rectum)** | | |  |
|  | Hepatocytes | | - |  | Crypts, epithelial cells# | | ++ |
|  | Gall bladder | |  |  | Caecal Peyer's patch | | ++ |
|  |  | Mucosa | + | **Female reproductive organs** | | |  |
|  |  | Bile canaliculi | ++ |  | Uterus and vaginal endometrium | | + |
|  |  | Bile ductules and ducts | + |  | Cervix endometrium | | - |
| **Skin** | | |  |  | Oviducts mucosal epithelium | |  |
|  | Epidermal Kertinocytes | | - |  |  | Ampullae | +++ |
|  | Hair bulb and shaft | | - |  |  | Isthmus of ampullae and uterus | + |
|  | Hair root and sheath | | - |  |  | Infundibulum | + |
|  | Sebaceous glands | | ++ |  | Ovaries** | |  |
|  | Sweat glands | |  |  |  | Germinal epithelium | + |
|  |  | Acinar cells | ++ |  |  | Ovarian bursa and follicles | - |
|  |  | Duct cells | +++ | **Thymus** | | |  |
|  |  | Myoepithelial cells | - |  | Medullary thymic epithelium | | ++ |
| **Brain**‡ | | |  |  | Cortical thymic epithelium | | + |
|  | Neurons | | - | **Connective, adipose and muscular tissues; skeletal bones** | | | - |
|  | Glia cells | | - |  |  |  |  |
|  | Brain ependymal cells | | - | **Lymphoid organs (lymph nodes, spleen, Peyer's patch follicles)** | | | (+) |
|  | Choroid plexus ependymal cells | | ++ |  |  |  |  |
|  |  |  |  |  | Non-identified signals | |  |

**Supplementary Table S2. LSFM-based biodistribution scoring of anti-EpCAM (G8.8R) antibody binding after i.v. administration (20 µg, 24 h) based on fluorescence intensity levels.**

*****Cortical, medullary, papillary ducts (ducts of Bellini). Associated intercalated cells showed increased binding. Binding generally higher for cortical nephrons than juxtamedullary nephrons. **†**Individual structures in the medulla and pelvis not identified due to lack of contrast.  **‡**Brains were not precleared and dehydrated/delipidated using MeOH/DCM, which may have affected fluorescence signals differently than preclearing with EtOH-dehydration (as conducted for all other tissues)  **§** Glandular stomach highly heterogeneous binding pronounced near limiting ridge, lesser curvature  **||** Binding levels and pattern equal along duodenum, jejunum and ileum.
**¶** Signal pattern in lymphoid organs was non-polarized and likely originates from immunogenicity of the antibody and corresponding reactions of the hosts immune system.
**#** General gradient of binding pronounced towards crypt base/decreased towards luminal surface.
******We detected an unidentified spherical structure within the ovarian bursa that was highly positive but could not be allocated to any defined ovarian cell type (compare Suppl. Fig. S14E and F).


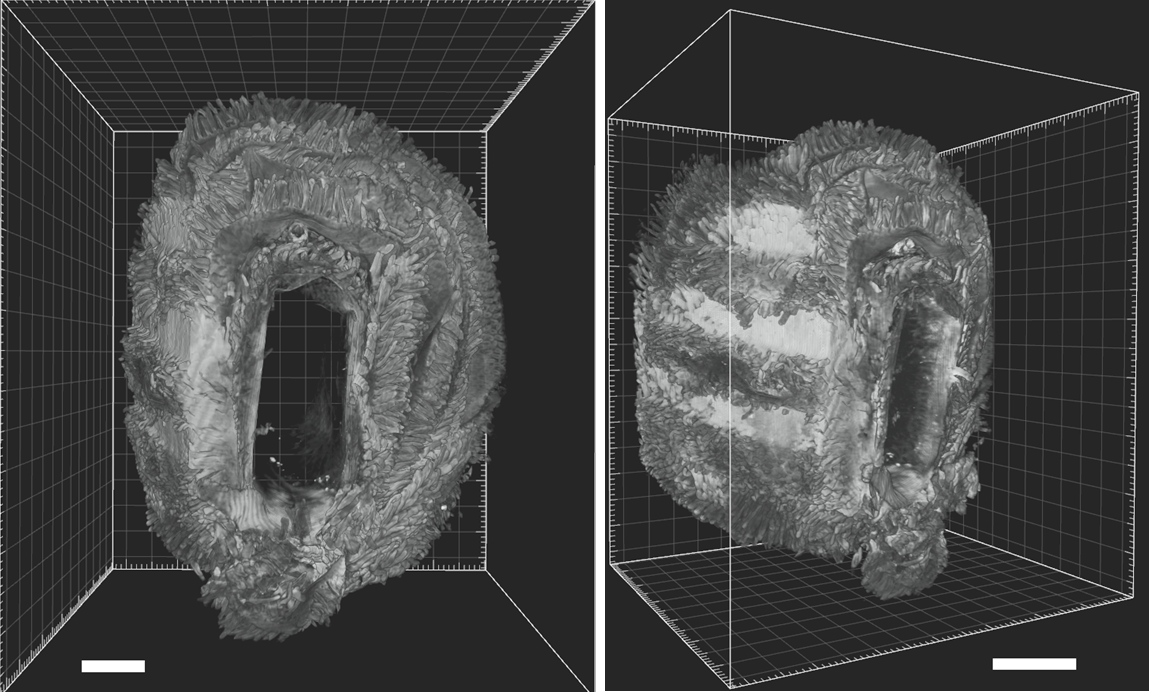
**Supplementary Figure S5. Deformation artifact of a 3D-Swiss Roll of the small intestine through contact with a processing cassette during fixation.** **(A)** Top view of a specimen of the small intestine (SI 1) that was squeezed and flattened (left side) by being pressed to the surface of a sample-processing cassette (arrows). **(B)** Side view of the same specimen with visible slits of the histology cassette imprinted in the tissue. Scale bars = 1 cm.

A

B


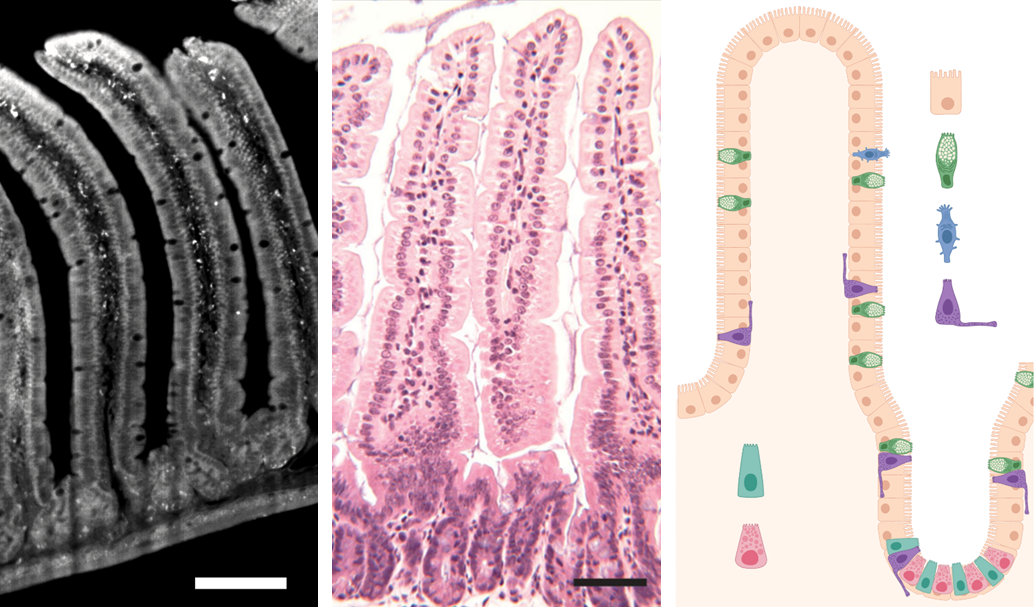
**Supplementary Figure S6.** **Light Sheet Fluorescence Microscopy (LSFM) enables analysis of microanatomical features and identification of cell types.** (**A**) Autofluorescence (grey) in a single high-resolution image of an LSFM scan of a 3D-Swiss Roll of the small intestine showing intact microanatomical tissue features. (**B**) Photomicrograph of a physical section of the small intestine stained with hematoxylin and eosin (H&E) as shown for a standard preparation technique (*49*). (**C**) Diagram of the murine small intestine depicting one villus with crypt and intestinal cell types. Enterocyte (e), nuclei (n), goblet cell (g), tuft cell (t), enteroendocrine cell (ee), stem cell (s), Paneth cell (p), crypts of Lieberkühn (c), tela submucosa (ts), tunica muscularis (tm). Scale bars = 100 µm.

(ee)

(p)

(s)

(p)

(c)

(t)

(g)

(e)

(g)

(tm)

(ts)

(c)

(n)

(e)

C

B

A

**
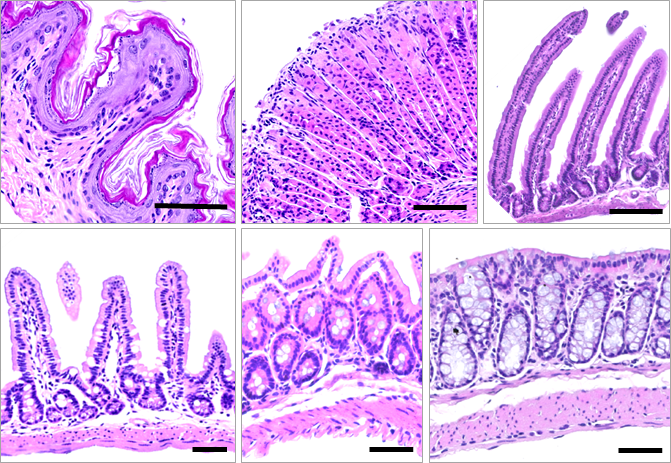
Supplementary Figure S7. Physical sections of the GIT stained with H&E following 3D-Swiss Roll processing, dehydration, clearing and LSFM imaging. (A)** Forestomach; **(B)** Glandular stomach fundus; **(C)** Duodenum; **(D)** Ileum; **(E)** Caecum; **(F)** Colon. All specimens retained regular microanatomical features and staining characteristics after previous processing as 3D-Swiss Rolls, tissue clearing and LSFM imaging. Scale bars = 50 µm.

D

A

F

E

C

B

**
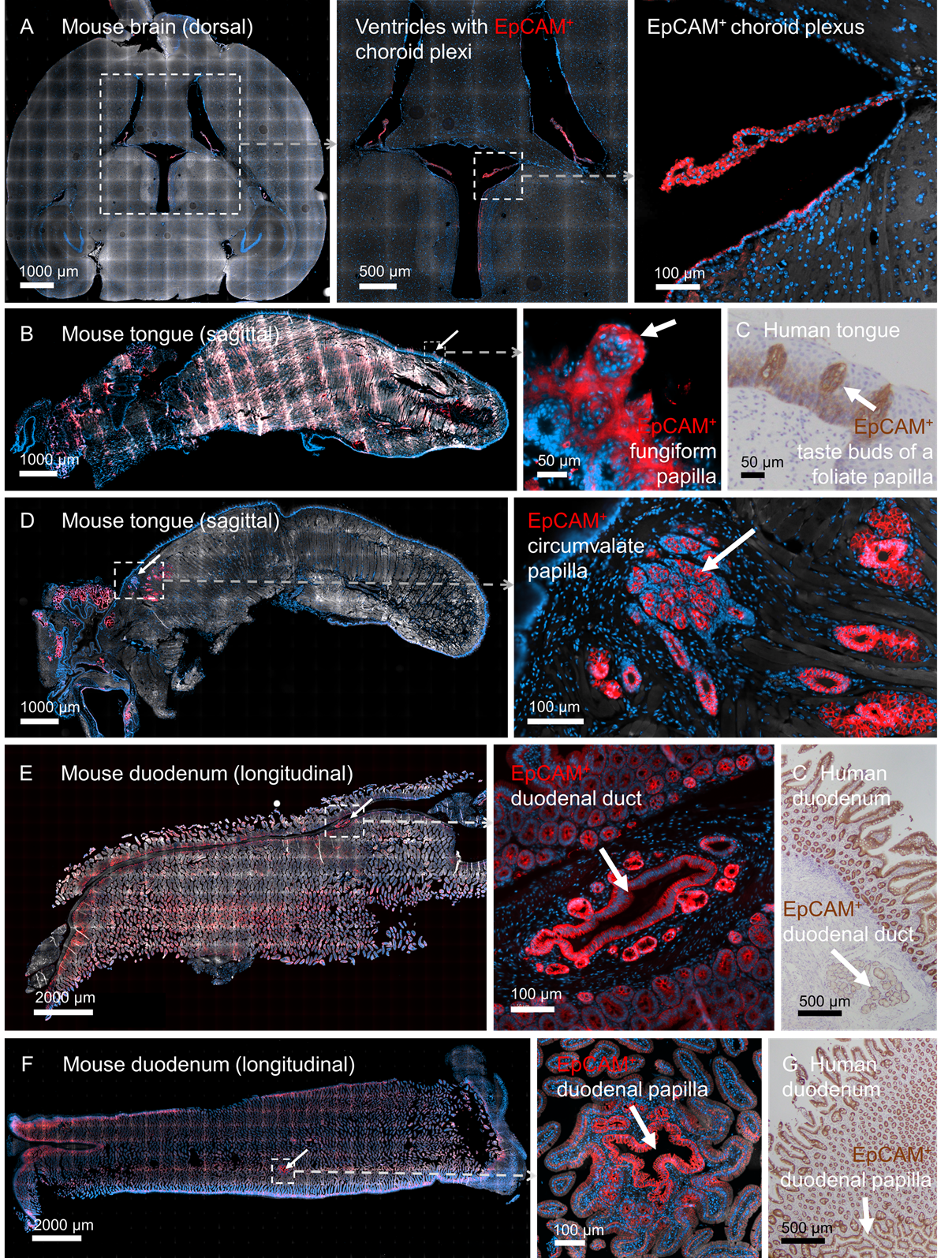

Supplementary Figure S8. Formalin-fixed paraffin-embedded tissue (FFPE) sections of murine and human tissues confirm novel binding sites of EpCAM-targeted antibodies as discovered by LSFM imaging. (A)** Mouse brain with EpCAM^+^ choroid plexi inside all ventricles. **(B-D)** Mouse and human tongue with EpCAM^+^ taste buds of fungiform and foliate gustatory papillae. **(E-F)** Mouse and human sections of the duodenum with EpCAM+ duodenal ducts and papillae.

**
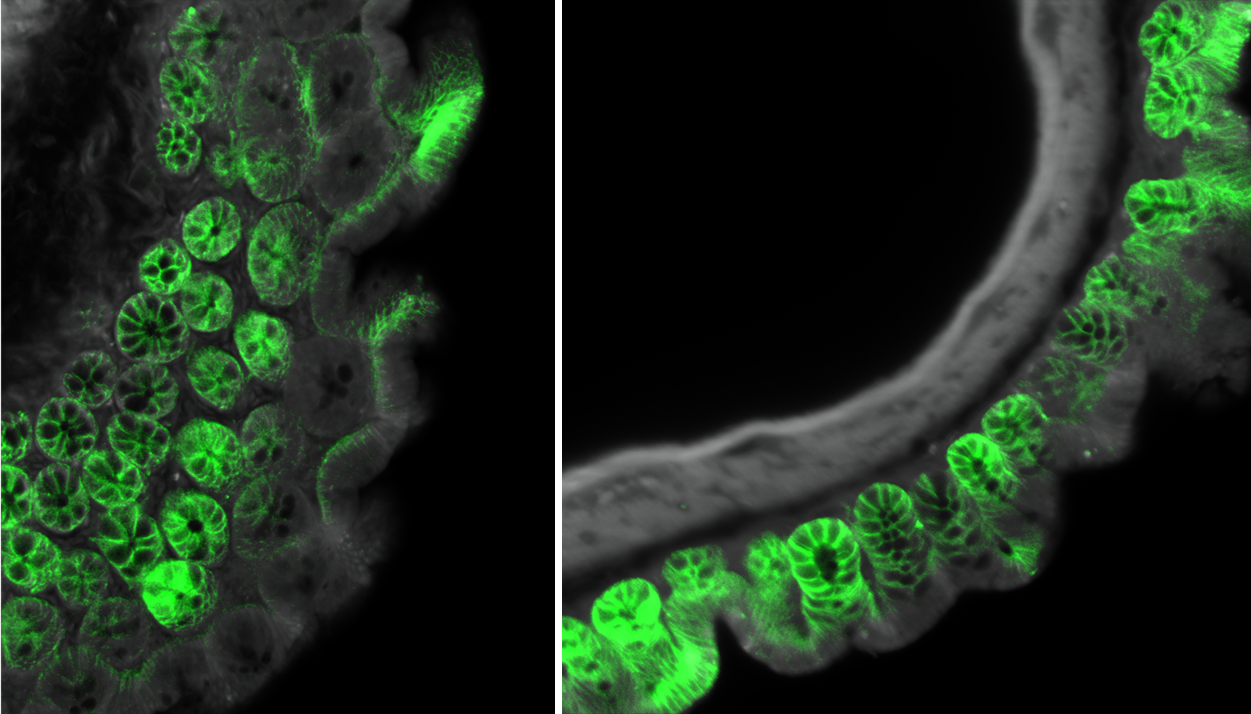
Supplementary Figure S9. Single LSFM images reveal basolateral EpCAM-AF750-binding patterns to epithelia. (A)** Caecum and **(B)** colon with polarized binding patterns of the antibody to lateral (dashed arrows) and basal (arrows) membranes of epithelial enterocytes. This binding pattern was observed for all normal simple epithelia throughout the body. *Luminal side of the tissue is indicated by an asterisk. Scale bars = 50 µm.

*****

*****

*****

*****

B

A

**
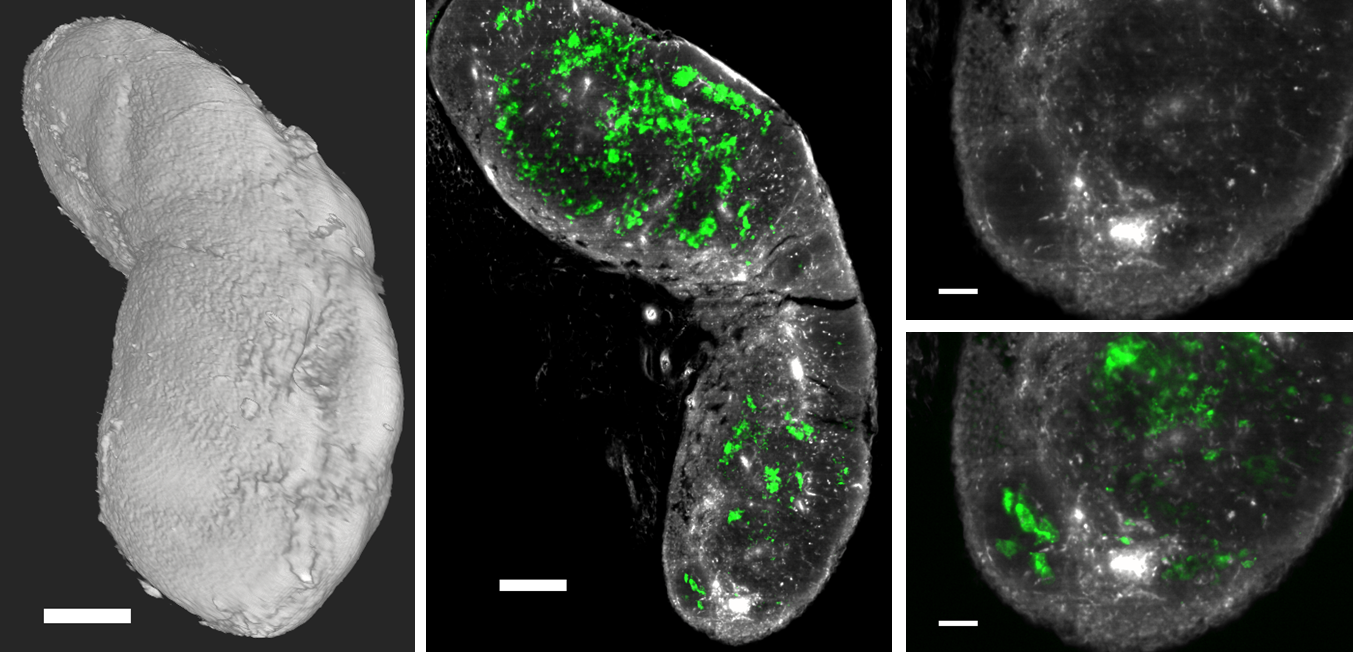
**

(C, D)

D

HIL

BCF

C

B

A

**Supplementary Figure S10. Anti-EpCAM-AF750 antibody staining in lymph nodes.** **(A)** 3D-Surface rendering and **(B)** maximum intensity projection of a z = 100 µm virtual section (MIP_100µm_) of a single inguinal lymph node at approximately half width (central) depicting general anatomy (grey) and antibody binding (green). **(C, D)** Single digital sections of the area indicated in image (B). Hilus (HIL), B cell follicle (BCF, *bona fide*). Scale bars = 200 µm (A, B), 50 µm (C, D).


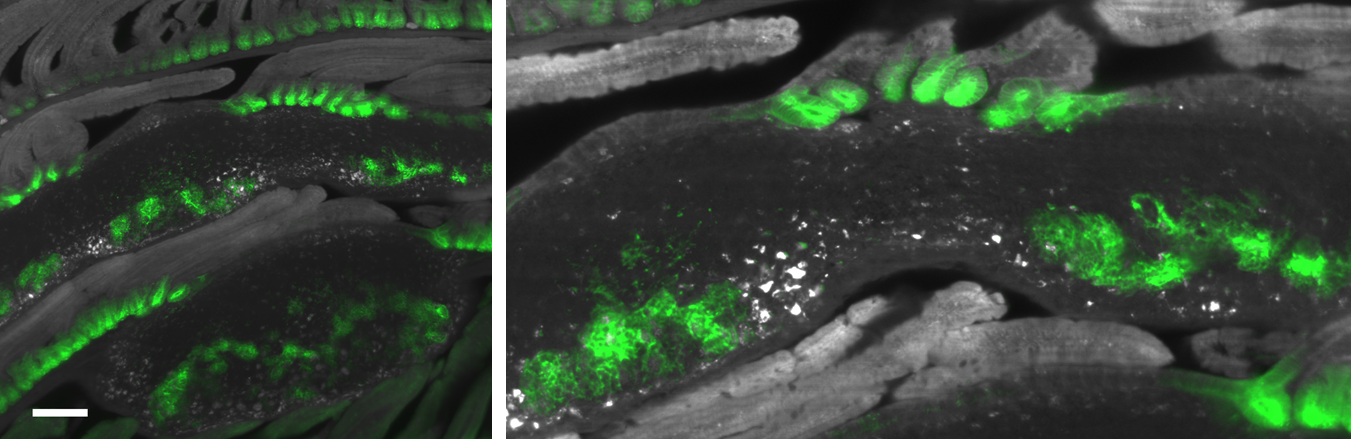


PP

B

BCF

IFR

SED

DE

C (SI)

SED

A

PP

**Supplementary Figure S11. Anti-EpCAM-AF750 antibody binding in Peyer's patches (PP).** **(A)** MIP_100µm_ of two PPs (encircled) in consecutive layers of the 3D-Swiss Roll of the small intestine. Note the increased antibody binding to crypts of the small intestine C(SI) between two follicles of one PP. DE = dome epithelium. **(B)** MIP_100µm_ of the upper PP (encircled) depicted in image (A), B cell follicles (BCF*, bona fide*) with antibody binding (green). Note the decreasing signal intensity at the DE. The subepithelial dome (SED) and interfollicular regions (IFR) were always excluded from antibody binding. Scale bars = 200 µm (A), 50 µm (B).


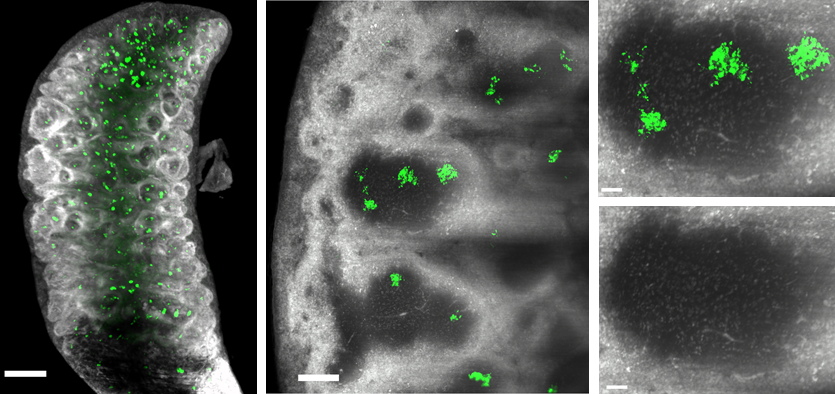


(B)

C

B

(C, D)

A

D

WP

RP

**Supplementary Figure S12. Anti-EpCAM-AF750 antibody binding in the spleen. (A)** Maximum intensity projection (MIP_WHOLE_) of the entire spleen anatomy (grey) and antibody binding (green). Note melanosis on the bottom left of the organ (arrow). **(B)** MIP_100µm_ of the region indicated in image (A) depicting several white pulp areas (WP, darker spots in autofluorescence, grey), red pulp (RP, bright areas) and the capsule (edge). **(C, D)** MIP_100µm_ of the region indicated in image (B). Note that antibody accumulations (green) do not correspond to any distinct structures in the autofluorescence channel (encircled, D). Scale bars = 1000 µm (A), 150 µm (B), 30 µm (C, D).


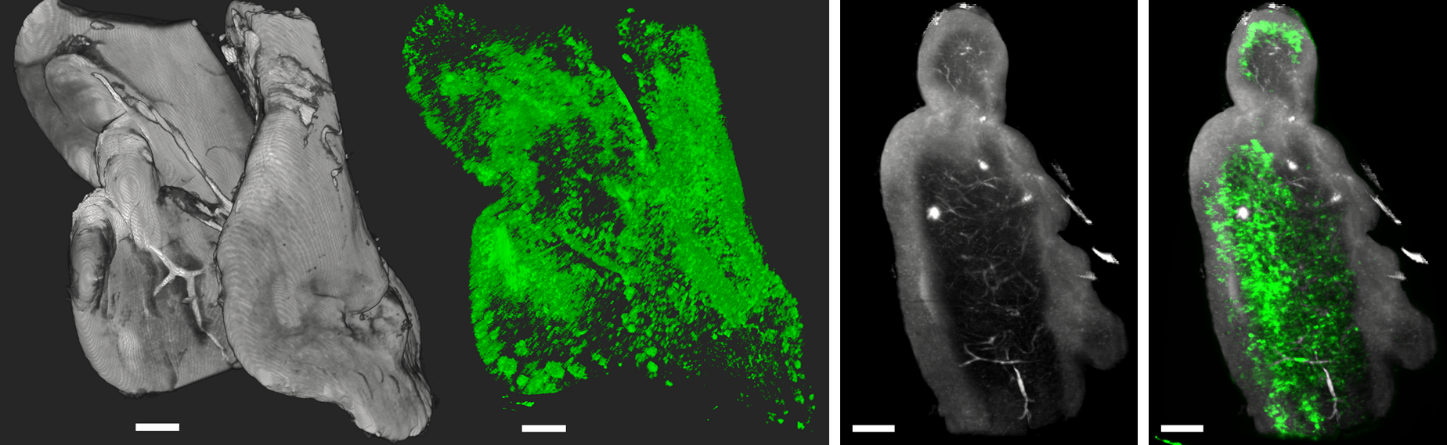


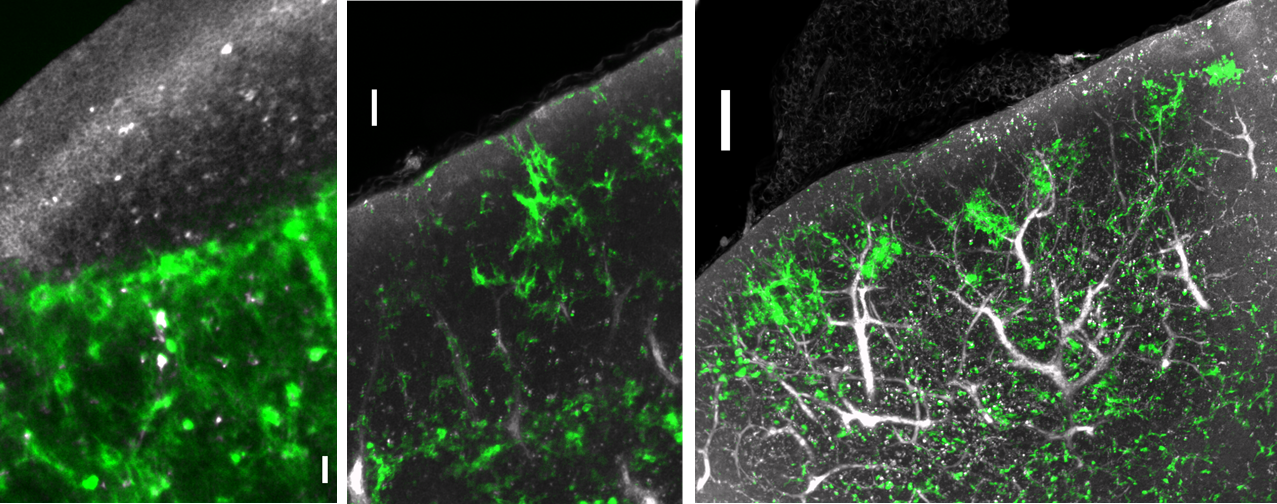
**Supplementary Figure S13. Anti-EpCAM-AF750 antibody binding in in the thymus. (A)** Surface rendering of both lobes (each encircled) of the thymus (left, grey) and respective antibody binding (right, green). **(B)**MIP_200µm_ of the left lobe anatomy (grey, Ch2) and **(C)** anatomy and antibody tissue binding as overlay (green, Ch5). **(D)** Single LSFM image depicting the interface of cortex (Cor) and medulla (Med) with locally restricted antibody binding in the medulla. Arrows indicate some of many speckles detected within the overall mesh-like binding pattern. **(E)** Another region of the same specimen showing binding also in the cortex. **(F)** MIP_100µm_ of the thymus showing focally increased binding in distinct areas (arrows). Scale bars = 250 µm (A-C), 40 µm (D), 50 µm (E), 100 µm (F).

F

Med

E

D

Cor

Cor

Med

A

B

Cor

C

**
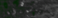

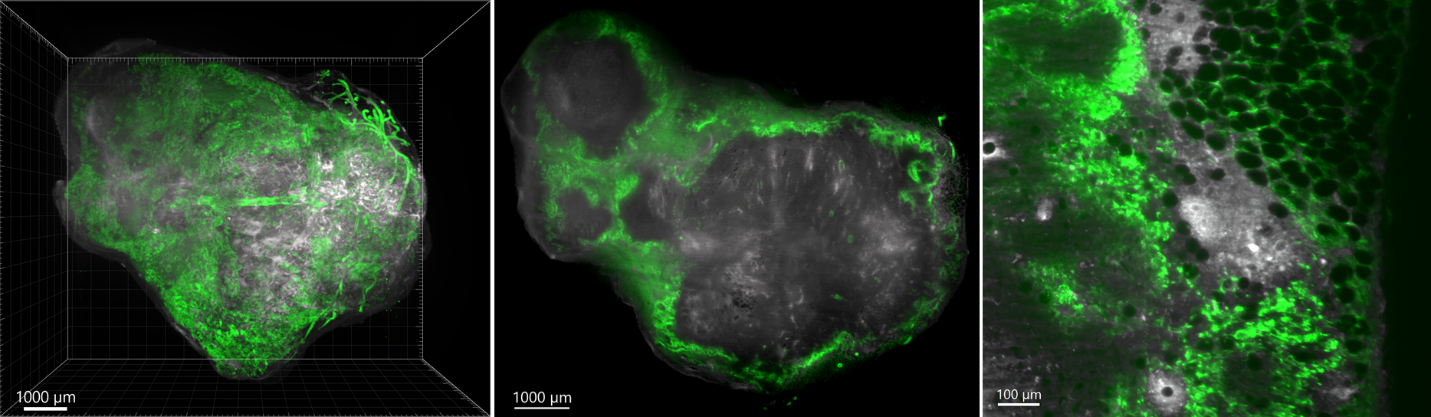
Supplementary Figure S14. Anti-EpCAM-AF750 antibody binding in the tumor. (A)** 3D-rendering of a subcutaneous tumor of a pancreatic cancer cell line (KPC-4662, green) **(B)** Single digital section (slice 527/661) of the tumor depicting highly heterogeneous binding of the antibody. **(C)** Higher magnification (10x) image of the region indicated in (B). Scale bars = 1000 µm (A, B), 100 µm (C)

B

(C)

C

A

**
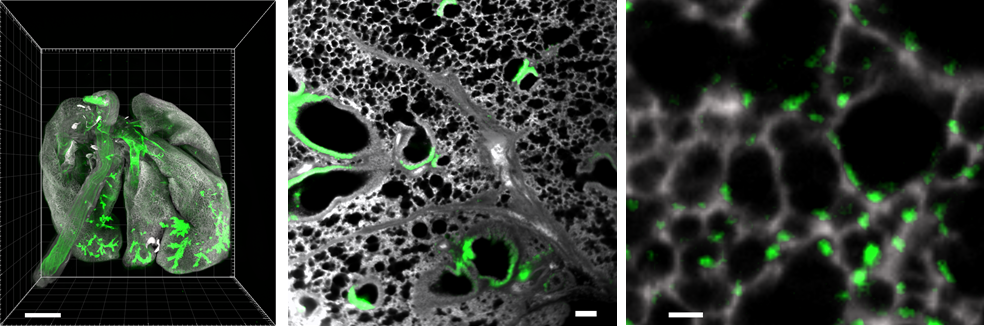
**
**Supplementary Figure S15. Anti-EpCAM-AF750 antibody binding in in the lower respiratory tract.** **(A)** Dorsal view of the gross lung anatomy (grey) and antibody binding (green). Left lobe (LL), superior right lobe (SRL), medial right lobe (MRL), Inferior right lobe (IRL), trachea (TR). View of post-caval lobe is obstructed. **(B)** Enlarged single slice view of the region indicated in (A) depicting several bronchioli (BR) and detected antibody (green). **(C)** Scattered localization of the antibody within the alveoli protruding into the alveolar space (arrows). Scale bars: 1000 µm (A), 200 µm (B) and 100 µm (C).

(B)

B

C

LL

TR

MRL

IRL

SRL

BR

A


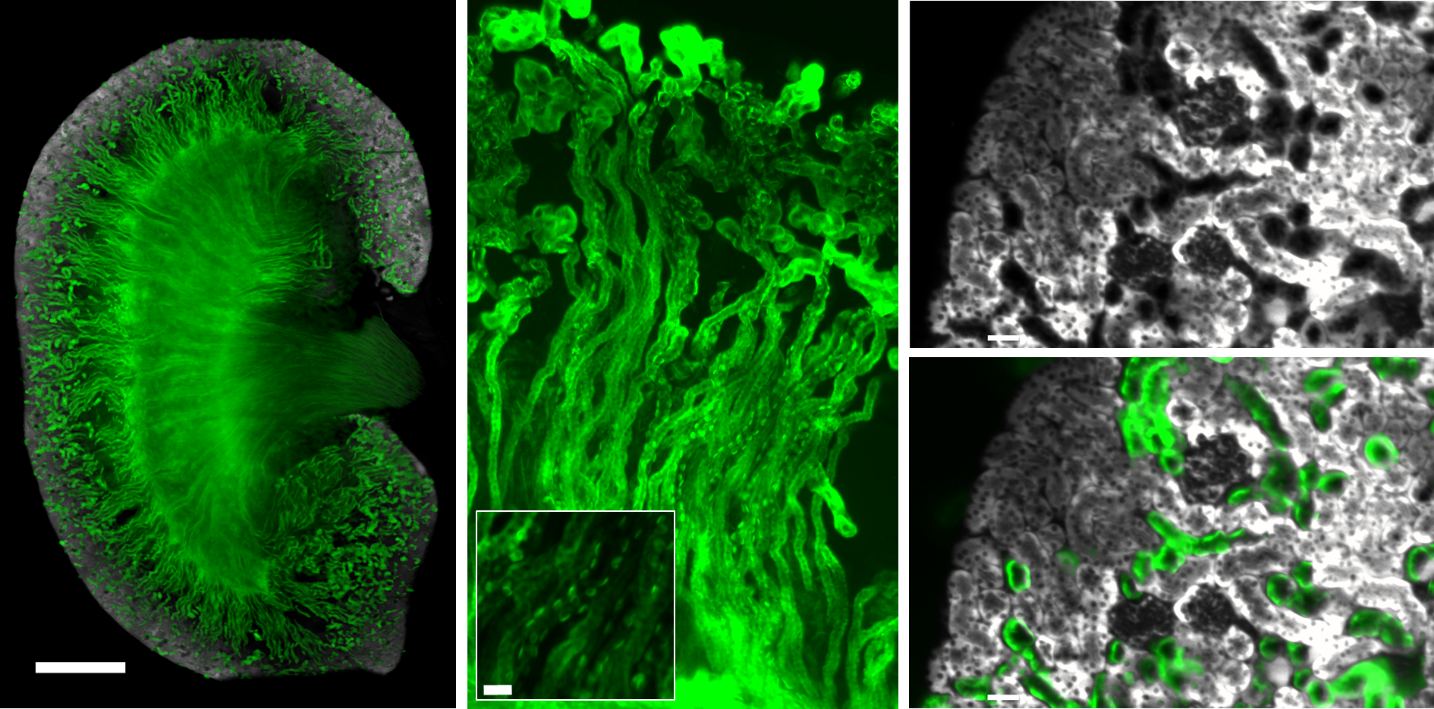


B

(C, D)

D

C

PCT

DCT

GLO

CD

DCT

PEL

MED

(B)

IC

COR

A

**Supplementary Figure SS16. Anti-EpCAM-AF750 antibody binding in the kidney. (A)** Maximum intensity projection of 50 µm tissue (MIP_50µm_) at the center of the kidney depicting its anatomy (grey) and bound anti-EPCAM antibody (green). Medulla (MED), Cortex (COR), Pelvis (PEL). **(B)**MIP_30µm_ of area indicated in (A) depicting only the antibody signal. Distal convoluted tubule (DC), proximal convoluted tubule (PC), collecting duct (CD), intercalated cell (IC). Scale bars: 2000 µm (A), 10 µm (B, box), 50 µm (B, C and D).

**
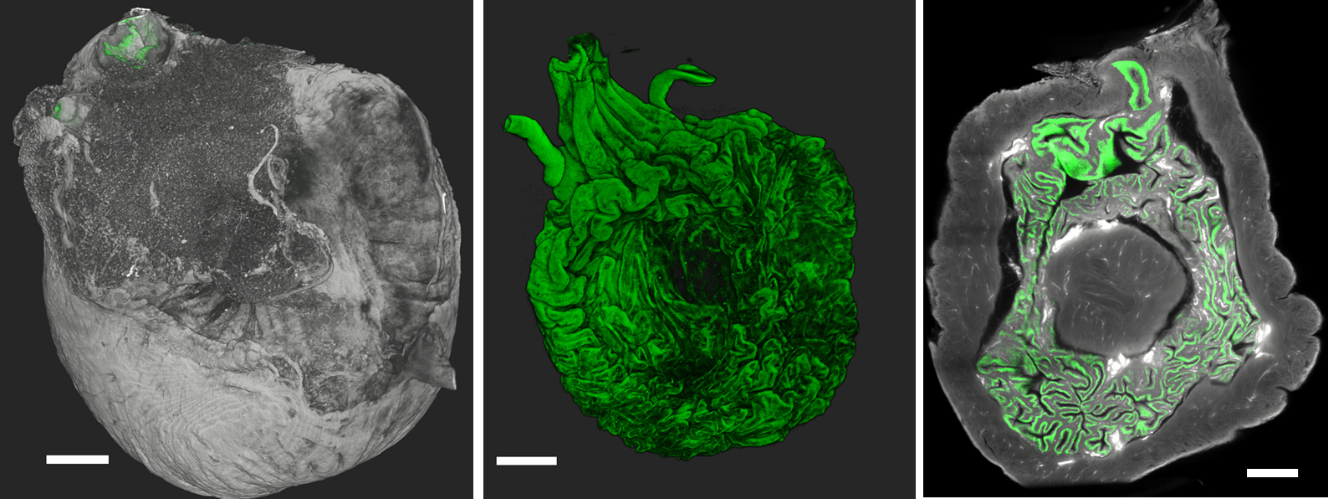
**

UR

UO

UR

UO

#### TS

TA

TM

#### UOP

C

B

A

TMu

**Supplementary Figure S17. Anti-EpCAM-AF750 antibody binding in the bladder (A)** Rendering of the entire bladder anatomy **(B)** Surface rendering of the antibody binding in the entire bladder. **(C)** Single LSFM image depicting the bladder anatomy (grey) and bound antibody (green). Urethra (U), urethral opening (UO), tunica adventitia (TA), tunica subserosa (TS), tunica muscularis (TM), Tunica mucosa (TMu). Scale bars = 400 µm.


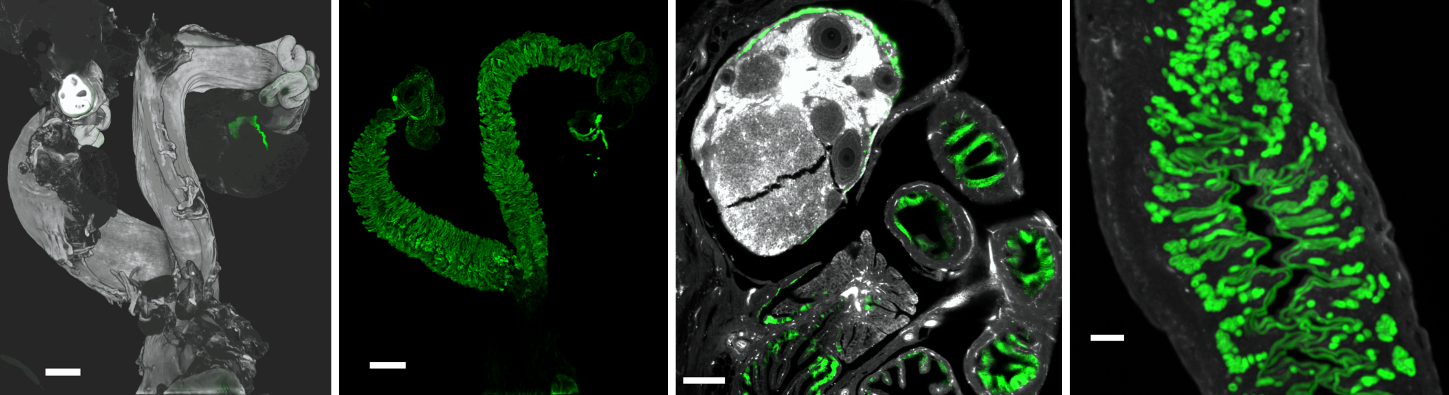


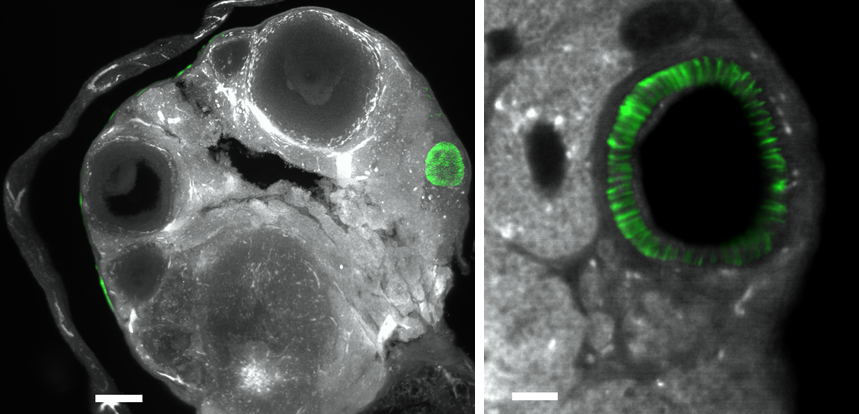


C

Amp

TSer

OB

(F)

F

E

(C)

(D)

OF

GE

Inf

TMuc

TMus

D

B

A

Cor

Ovi

Ova

**Supplementary Figure S18. Anti-EpCAM-AF750 antibody binding in the female reproductive organs**. **(A)** General morphology of the specimen (grey) and **(B)** overview of EpCAM tissue binding (green). Arrow indicates a spherical accumulation of antibody in one of the ovaries (see also E, F). **(C)** Single LSFM image of one of the ovaries and oviducts as indicated in (A). **(D)** Single LSFM image of one of the ampullae as indicated in image (B). Corpus (Cor), infundibulum (Inf), ovary (Ova), oviduct (Ovi), tunica mucosa (TMuc, endometrium), tunica muscularis (TMus), tunica serosa (TSer), ovarian follicle (OF), ovarian bursa (OB), germinal epithelium (GE), ampulla (Amp). **(E)** MIP_500µm_ of the left ovary showing antibody binding to the germinal epithelium (arrow) and an unidentified spherical object with very high antibody binding (boxed, and F). **(F)** Single Image as indicated in (E) showing lateral membranous binding. Scale bars = 1000 µm (A, B), 300 µm (C), 200 µm (D), 100 µm (E), 30 µm (F).

**
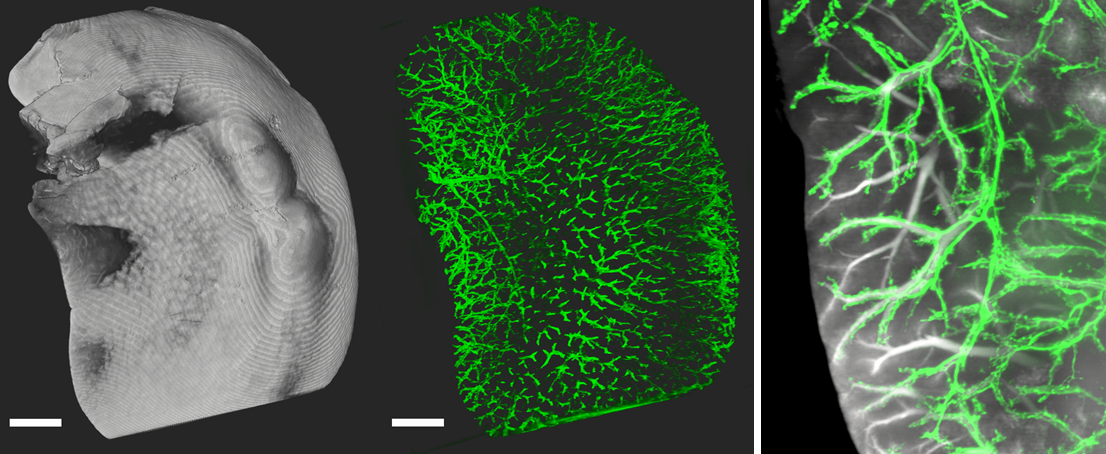
**

B

LHD

(B)

(B)

A


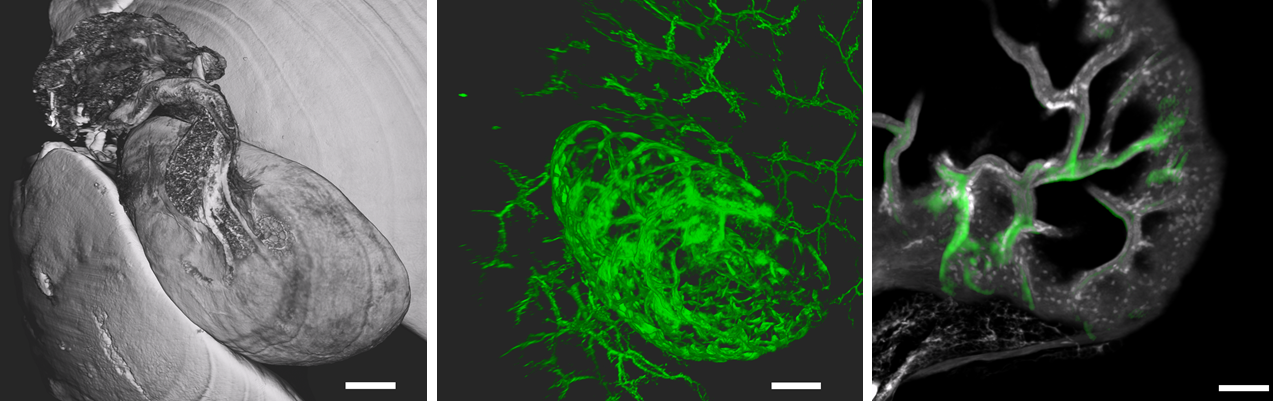


E

LP

TS

RML

LML

TM

(E)

D

C

*****

GB

**Supplementary Figure S19. Anti-EpCAM-AF750 antibody binding in the liver and gall bladder.** **(A)** Ventral surface rendering of left liver lobe anatomy (left, grey) and MIP_WHOLE_ of antibody (right, green) bound to the entire biliary ductal tree with visible left hepatic duct (LHD).
**(B)** MIP_200µm_ overlay of both channels as indicated in (A). Antibody binding to biliary ducts was closely associated with blood vessels. **(C)** Surface rendering of the gall bladder (GB) anatomy *in situ* situated between left (LML) and right (RML) medial liver lobe. **(D)** Antibody binding as detected in (C) bound to the GB and biliary ducts in the adjacent liver lobes. **(E)** MIP_50µm_ of the gall bladder fundus as indicated in (D) with visible lamina propria (LP), tunica serosa (TS) and tunica mucosa (TM). *asterisk indicates luminal side. Scale bars = 2000 µm (A), 200 µm (B), 300 µm (C, D), 50 µm (E).

**
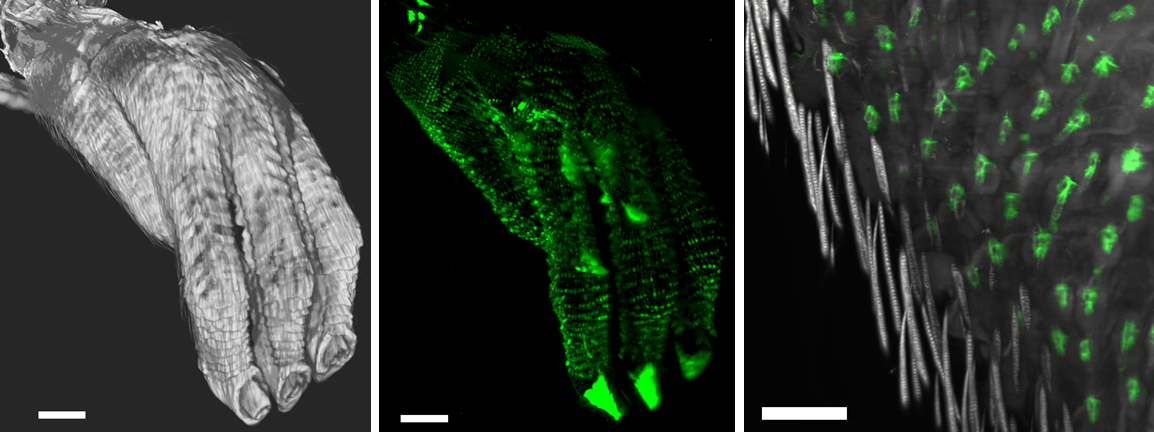
**

C

B

A

(C)

**Supplementary Figure S20. Anti-EpCAM-AF750 antibody binding at hair follicles and sweat glands in the paw.** **(A)** 3D-surface rendering of the anatomy of the left paw. **(B)** MIP_WHOLE_ of the antibody binding with increased binding to all footpads (arrows). **(C)** MIP_50µm_ of the general tissue anatomy (grey) and bound antibody as indicated in (B). Arrow indicates one single hair follicle with antibody bound at sebaceous glands. Scale bars = 1000 µm (A, B), 200 µm (C).


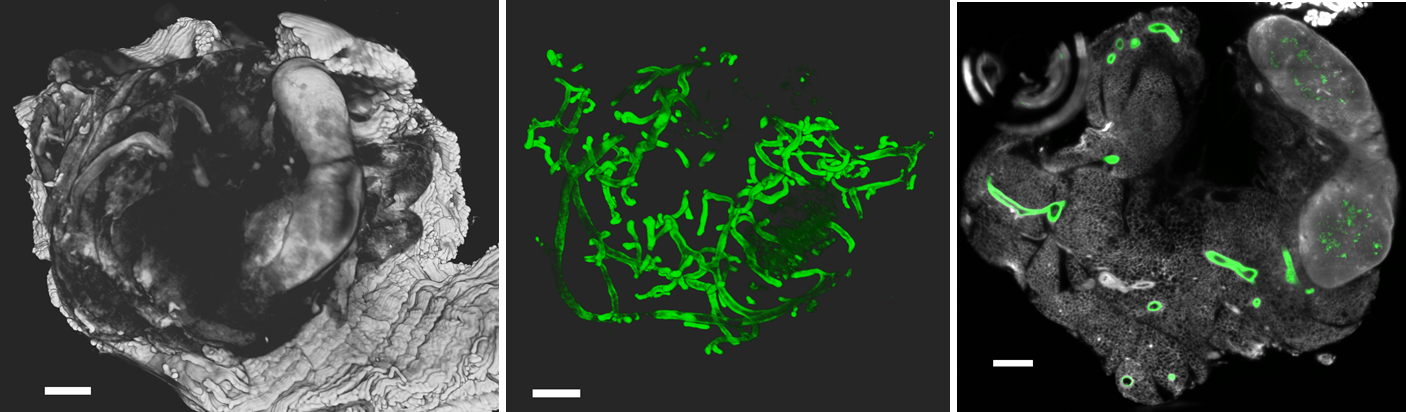


LN

C

B

A

**Supplementary Figure S21. Anti-EpCAM-AF750 antibody binding in the mammary glands.** **(A)** 3D-surface rendering of a mammary gland embedded in muscular and adipose tissue. **(B)** 3D-surface rendering of the bound antibody at the lactiferous ducts. **(C)** Single slice view depicting antibody binding (green) within the mammary gland tissue at the epithelium of the ducts (arrows). Note the lymph node (LN) that was by-sampled together with the glandular tissue. Scale bars = 200 µm.

**
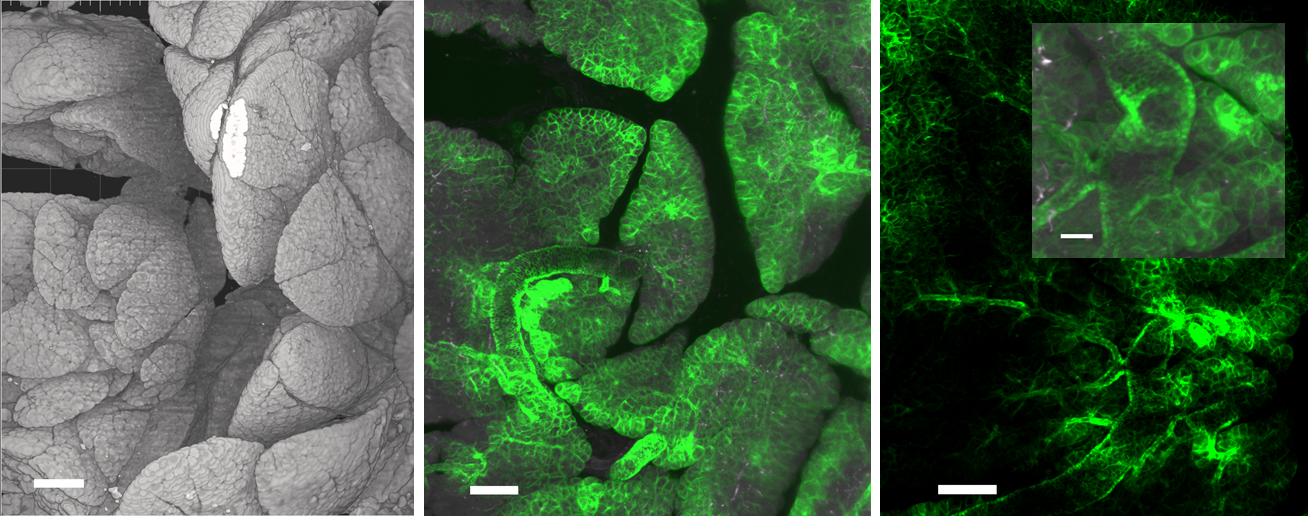
Supplementary Figure S22. Anti-EpCAM-AF750 antibody binding in the pancreas (A)** Partial 3D-surface rendering of the gastric division of the pancreas. **(B)** MIP_100µm_ of the area indicated in (A) showing the general tissue anatomy (grey) and antibody binding (green). Binding was restricted to membranes of acini (AC) and epithelia of pancreatic ducts (PD). **(C)** MIP_100µm_ of the same area as image (B) at a different depth with Ch2 (autofluorescence) turned off. Enlargement depicts a branched pancreatic duct (*PD*) with antibody bound evenly distributed to the entire epithelium and surrounding positive acinar cells (*ACs*). Scale bars = 200 µm (A and B), 50 µm (C).

C

B

A

(B, C)

PD

ACs

PD

AC
